# Highly Active 2D Layered MoS_**2**_**-**rGO Hybrids for Energy Conversion and Storage Applications

**DOI:** 10.1038/s41598-017-08677-5

**Published:** 2017-08-21

**Authors:** Swagatika Kamila, Bishnupad Mohanty, Aneeya K. Samantara, Puspendu Guha, Arnab Ghosh, Bijayalaxmi Jena, Parlapalli V. Satyam, B. K. Mishra, Bikash Kumar Jena

**Affiliations:** 1 CSIR-Institute of Minerals and Materials Technology, Bhubaneswar, 751013 India; 2 Academy of Scientific & Innovative Research, New Delhi, 110001 India; 3 Institute of Physics, Bhubaneswar-751005, Bhubaneswar, India; 4 Homi Bhabha National Institute, Training School Complex, Anushakti Nagar, Mumbai, 400085 India; 5 Department of Physics, Indian Institute of Technology Kharagpur, Kharagpur, 721302 India; 6 Department of Chemistry, Utkal University, Bhubaneswar, 751004 Odisha India

## Abstract

The development of efficient materials for the generation and storage of renewable energy is now an urgent task for future energy demand. In this report, molybdenum disulphide hollow sphere (MoS_2_-HS) and its reduced graphene oxide hybrid (rGO/MoS_2_-S) have been synthesized and explored for energy generation and storage applications. The surface morphology, crystallinity and elemental composition of the as-synthesized materials have been thoroughly analysed. Inspired by the fascinating morphology of the MoS_2_-HS and rGO/MoS_2_-S materials, the electrochemical performance towards hydrogen evolution and supercapacitor has been demonstrated. The rGO/MoS_2_-S shows enhanced gravimetric capacitance values (318 ± 14 Fg^−1^) with higher specific energy/power outputs (44.1 ± 2.1 Whkg^−1^ and 159.16 ± 7.0 Wkg^−1^) and better cyclic performances (82 ± 0.95% even after 5000 cycles). Further, a prototype of the supercapacitor in a coin cell configuration has been fabricated and demonstrated towards powering a LED. The unique balance of exposed edge site and electrical conductivity of rGO/MoS_2_-S shows remarkably superior HER performances with lower onset over potential (0.16 ± 0.05 V), lower Tafel slope (75 ± 4 mVdec^−1^), higher exchange current density (0.072 ± 0.023 mAcm^−2^) and higher TOF (1.47 ± 0.085 s^−1^) values. The dual performance of the rGO/MoS_2_-S substantiates the promising application for hydrogen generation and supercapacitor application of interest.

## Introduction

The continuous and widespread use of fossil fuels has led to increased pollution and ecological problems^1^. Today these problems have resulted in unrepairable damage and the crisis is such that almost all countries have started switching to alternate energy resources^2, 3^. In this regard, substantial research efforts have been directed to the development of efficient and eco-friendly technologies for the production of energy conversion and storage devices^4^. Out of others, the energy from H_2_ has been pursued as a clean and alternative source of fossil fuel. The generation of H_2_ from water is the most important method which stands as a potential alternative to our energy demands^5^. The photochemical, photoelectrochemical and electrochemical methods have been broadly used for splitting of water^6, 7^. The electrochemical method is widely adopted due to its higher efficiency, cost effectiveness, and easy instrumental setups. However, the efficient conversion of H_2_O to H_2_ strongly depends on the activity of the electrode materials. The Pt-based materials are observed to be the most active and benchmark electrocatalyst which catalyses the conversion process nearly at zero overpotential^8^. However, the high cost and scarcity of the material limit its use for rapid commercialisation. Therefore, it demands to develop an alternative, low cost, electrocatalytic active material from abundantly available precursors by an efficient process.

On the other hand, the supercapacitors (SCs) arouse substantial attention due to its ease of fabrication, low cost, negligible environmental concerns, higher power output and excellent cyclic performances over the traditional secondary batteries^9^. These important properties keep the supercapacitor in demand for use as portable storage systems in electronic devices^10, 11^. Among other components of a supercapacitor, the electrode material plays an important role to evaluate the charge storage performance. Therefore, the effort has been devoted to developing different electrode materials like pseudo-capacitive or electrical double layered capacitive (EDLC) materials^12^. Whereas the prominent redox properties of the pseudo-capacitive materials make them show higher capacitance values compared to the EDLCs^13^. Thus, substantial scientific interest focuses on the development of different pseudocapacitive based materials like metal oxides, sulphides, nitrides and so forth^14^. However, the amorphous RuO_2_ has found to be an efficient electrode material for supercapacitor performance study, but the high cost and limited existence in the earth crust restrict its commercial application^15, 16^. Therefore, substantial research efforts must be paid to find promising materials with high capacitance behaviour.

Though many of the pseudocapacitive materials have been developed, molybdenum disulphide nanostructures of various surface morphologies show excellent performances^17^. Not only the interesting 2D layered structure of MoS_2_ avails higher surface area for charge accumulation but also the ion diffusion at the layer interfaces provides the redox properties^18, 19^. The combined effect of both these EDLCs and pseudocapacitive behaviour make the MoS_2_ as a suitable material for SC study. However, the lower conductivity and possible aggregation hinder the charge storage performance of the MoS_2_ demanding some alternative way of preparation^20^. The recent studies focus on the substrate mediated synthesis of MoS_2_ and their hybrids by mixing with the polymers, carbon nanotubes and their mixtures and explored their activities towards the SC and HER^21–23^. But the multi-step process and cost of the precursor materials demands to prepare MoS_2_ based materials in an alternative way using low-cost starting materials. Further, MoS_2_ is a nonprecious HER electrocatalyst with higher activity and excellent stability in acid medium. The computational and experimental studies established that the atomic basal plane of MoS_2_ has less activity towards HER but the sulphur edge sites of MoS_2_ possess excellent catalytic activity towards HER^24, 25^. Therefore, different strategies have been employed to increase the HER activity of MoS_2_ by increasing the active sites, the electrical contact of the sites^26, 27^, and exfoliation of layers to increase the surface area^28^. Also, the effort has been made to increase the activity of basal plane of MoS_2_^29^. The improvement of the electrical conductivity and exposure of more active sites plays an important role to improve the electrocatalytic performance of MoS_2_. However, the low intrinsic conductivity and restacking of the layered interfaces limit the performance of MoS_2_ for energy conversion and storage applications^30^.

In this work, MoS_2_ nanostructure and its hybrid with reduced graphene oxide have been successfully synthesized. The dual functional activity of the materials has been measured to ascertain the energy storage (supercapacitor) and energy conversion (HER) capacities. The hybridizations boosted the properties of individual components and thereby exhibited higher performance as a whole due to the synergistic contribution.

## Result and Discussion

**Figure 1:**
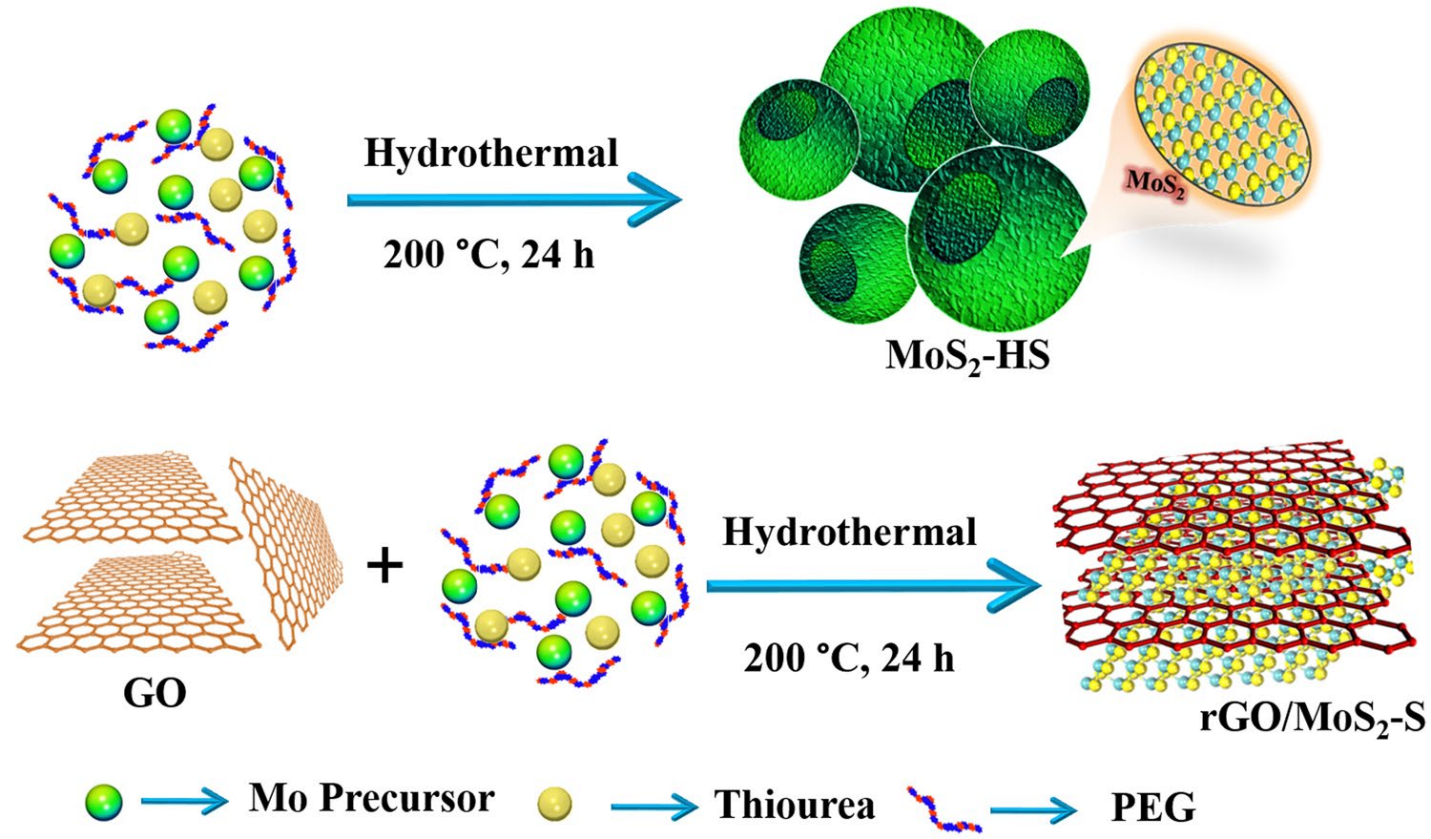
The schematic presentation for the synthesis of MoS_2_-HS and rGO/MoS_2_-S hybrids.

**Figure 2:**
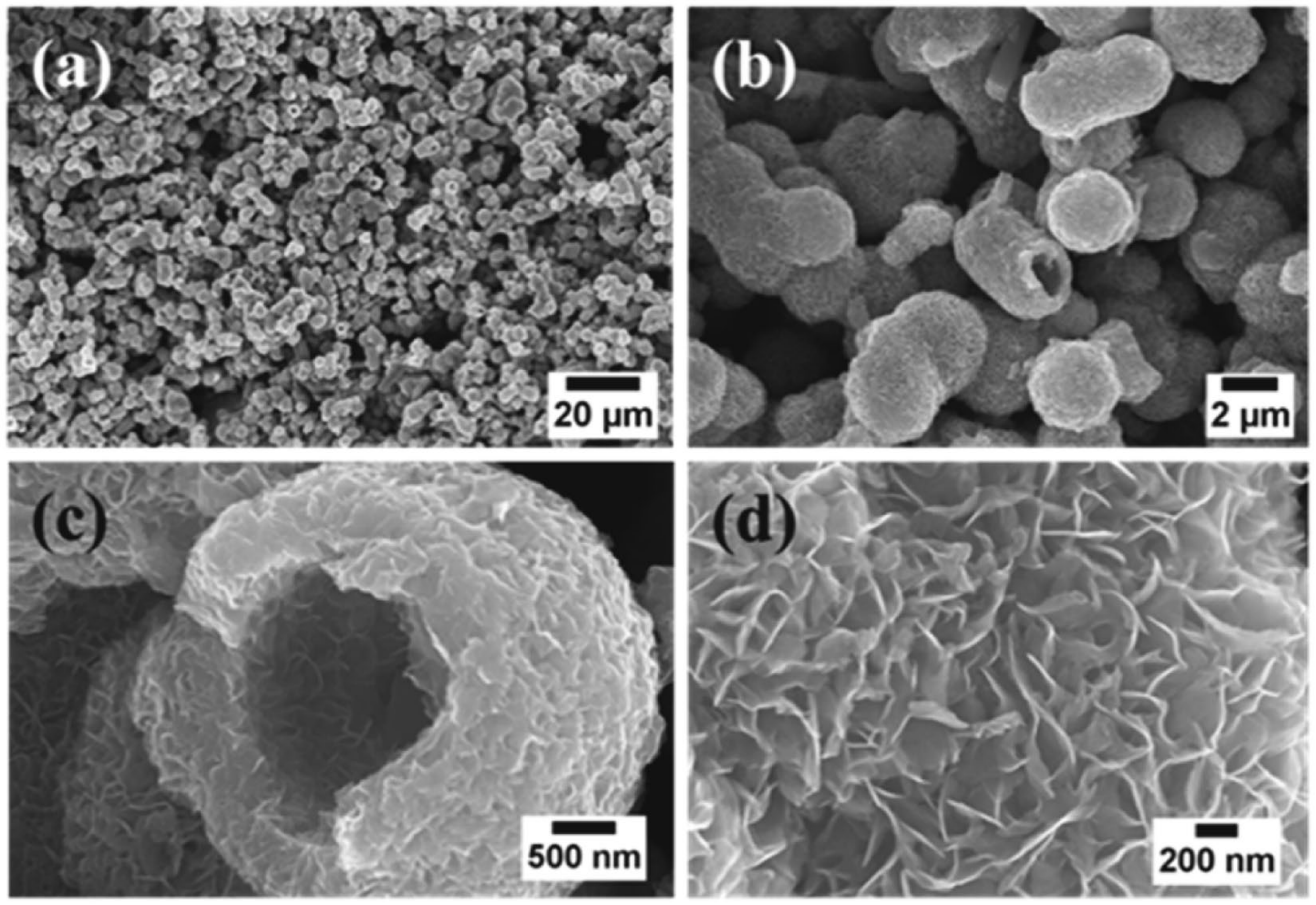
SEM image of MoS_2_-HS.

**Figure 3:**
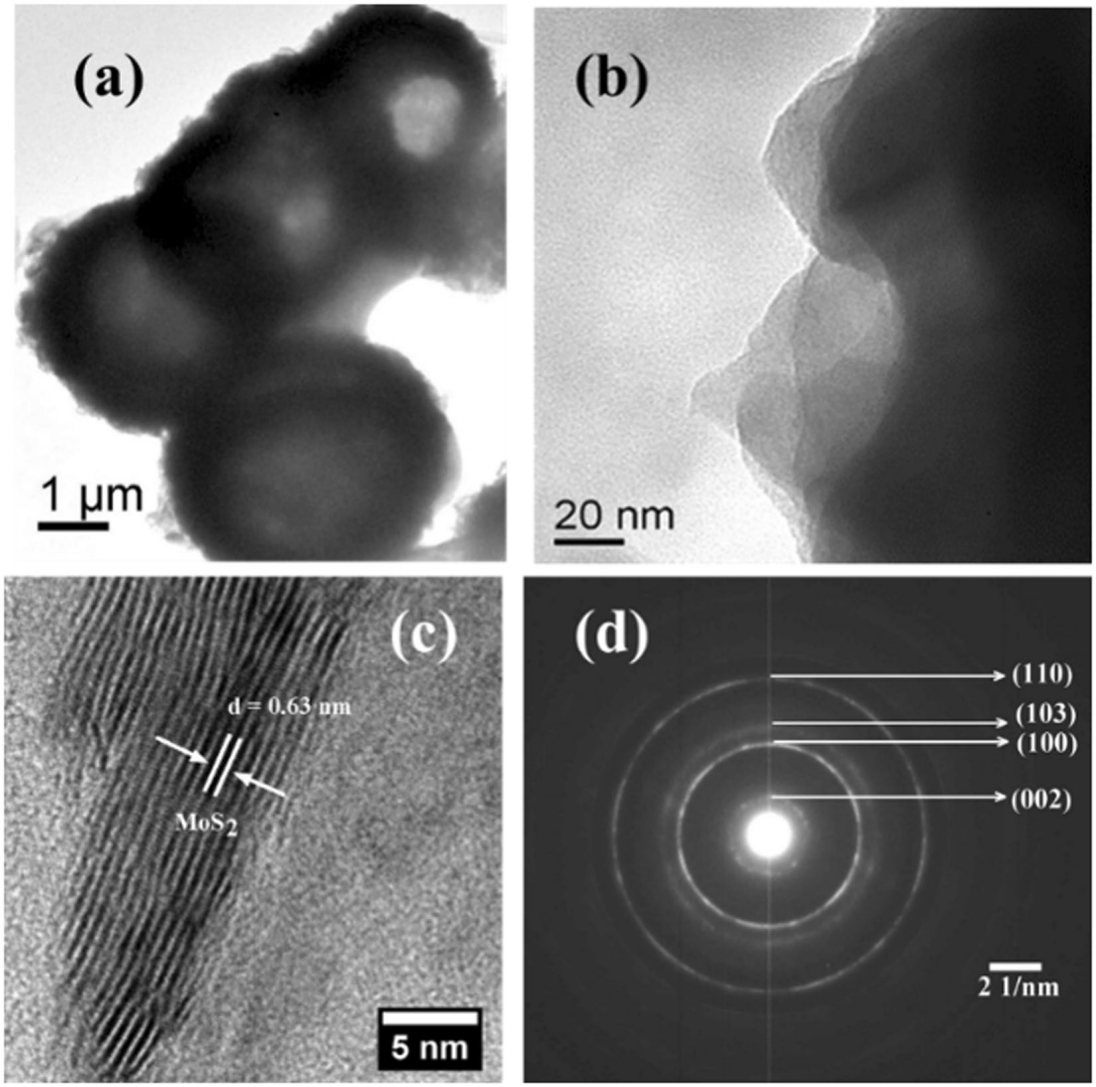
HRTEM image of MoS_2_-HS.

**Figure 4:**
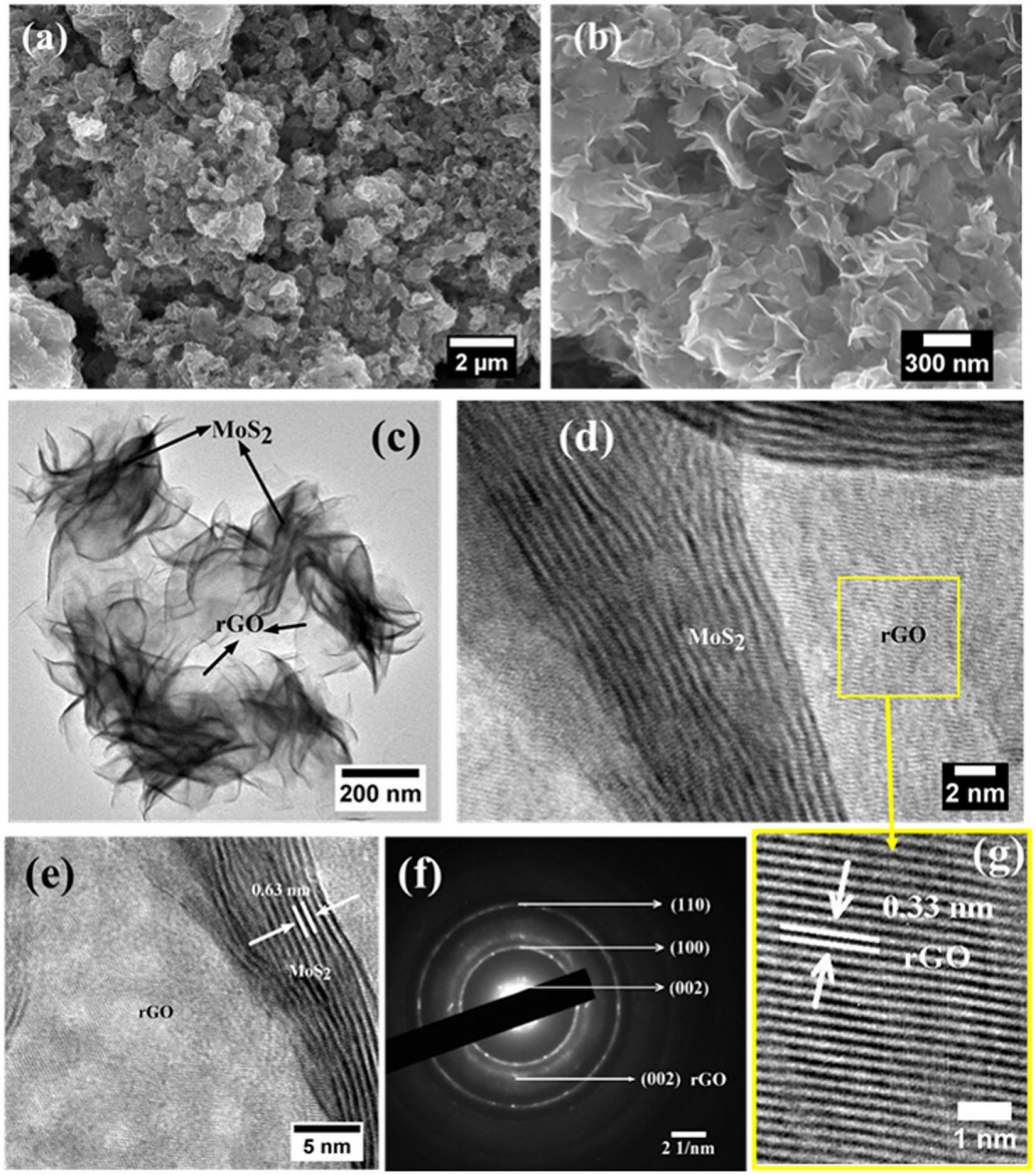
SEM (**a**,**b**) and HRTEM (**c**–**g**) image of the rGO/MoS_2_-S.

In this work, MoS_2_ nanostructure and its reduced graphene oxide hybrid have been synthesized by a facile hydrothermal synthesis method (Fig. 1). Here, ammonium heptamolybdate and thiourea were taken as the Mo and S source. The morphology of MoS_2_ was examined with the SEM measurements (Fig. 2). It can be seen that the MoS_2_ are in the form of hollow spheres (Fig. 2C). The high resolution of SEM image further reveals that the MoS_2_ hollow spheres (MoS_2_-HS) are constructed with layers of nanosheets (Fig. 2D). The elemental color mapping and EDS spectrum of as-synthesized MoS_2_-HS has been presented in Figure S1. Further, the morphology of the MoS_2_-HS was examined by transmission electron microscope (TEM) and the high-resolution TEM (HRTEM). It was observed that each of the hollow spheres constitutes well-arranged nanosheets throughout the surface (Fig. 3). The hi-resolution TEM image shows lattice fringes ≈0.63 nm indexed to the (002) plane of hexagonal phase of MoS_2_. The presence of diffraction ring patterns in the selected area electron diffraction (SAED) data supports the polycrystalline nature of the MoS_2_-HS. The MoS_2_ and its graphene hybrids were synthesized and examined with the SEM and TEM measurements (Fig. 4). As it can be seen from SEM images, the rGO/MoS_2_-S shows layers of MoS_2_ and rGO nanosheets embedded with each other. The distinct structures of MoS_2_-HS completely disappeared during the formation of rGO/MoS_2_-S. That may be due to the interaction between the functional groups of graphene oxide (GO) with Mo precursor which limits the growth of MoS_2_ layers to a unique hollow spherical structure as observed in the absence of GO. Interestingly, in the absence of GO, the MoS_2_ nanosheets were coalesced to form hollow sphere like morphology (vide infra). The elemental color mapping confirmed the presence of constituent elements (Mo, S, C and O) and clarified the compositional profile of the hybrids (Figure S2). Further, the EDS spectrum shows characteristic X-ray emission peaks for Mo, S, C and O (Figure S3). The TEM measurement reveals that the MoS_2_ and graphene sheets are well dispersed and embedded each other in rGO/MoS_2_-S (Fig. 4). It clearly demonstrates that the layer structure of MoS_2_ is uniformly distributed in the rGO sheet. The HRTEM image reveals the crystal lattice structure of MoS_2_ and rGO in rGO/MoS_2_-S. The interlayer spacing of MoS_2_ and rGO in the hybrid were estimated to be 0.63 nm and 0.33 nm respectively, which can be indexed to their (002) lattice planes (Fig. 4e and g). The SAED measurement supports the presence of distinct ring patterns indexed to the (002), (100) and (110) planes of MoS_2_ and (002) plane of rGO in the rGO/MoS_2_-S (Fig. 4f).

The XRD characterization was carried out to analyze the structural information for as-synthesized MoS_2_-HS and rGO/MoS_2_-S (Figure S4). In the case of MoS_2_-HS, the diffraction peaks were observed at 2θ of 14°, 32.4°, 35.4°, 43.3° and 57.4° corresponding to the (002), (100), (102), (006), (110) planes respectively (JCPDS 37–1492)^31^. The presence of (002) diffraction peak of MoS_2_-HS indicates that the stacking of monolayer MoS_2_ takes place along the c axis^32, 33^. In the case of rGO/MoS_2_-S, the (002) plane of MoS_2_ shifts towards 16.8° which can be estimated a decrease in the interlayer spacing value of 0.63 nm (002 of MoS_2_-HS) to 0.52 nm (002 of rGO/MoS_2_-S). That signifies the incorporation of graphene within the MoS_2_ crystal during the formation of rGO/MoS_2_-S^34^. However, the characteristic signal of rGO in rGO/MoS_2_-S was not observed in the XRD patterns. That can be attributed to the low content of rGO in the hybrid^35^.

**Figure 5:**
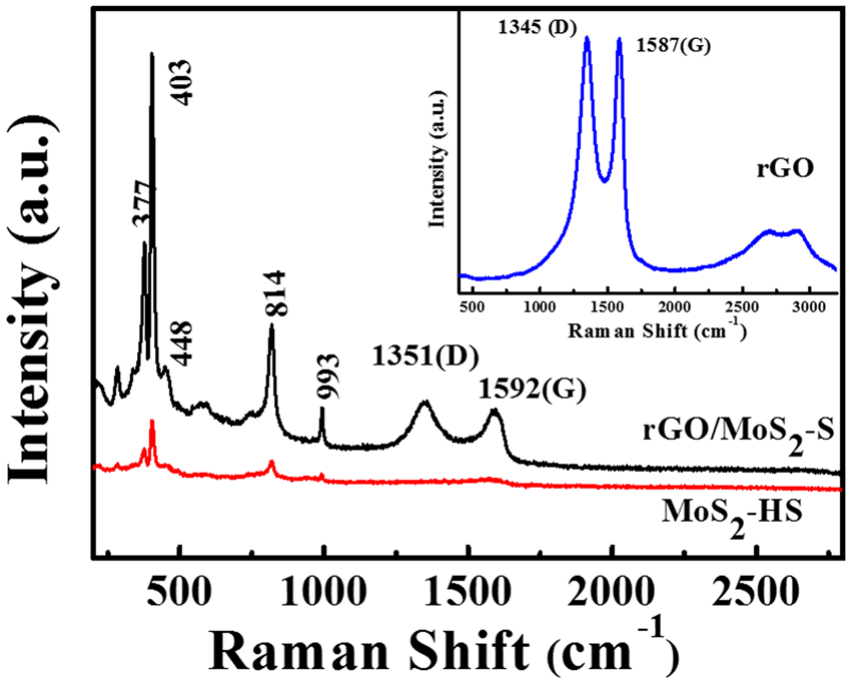
The Raman Spectra of MoS_2_-HS and rGO/MoS_2_-S hybrid.

The growth of rGO/MoS_2_-S was further investigated by the Raman spectroscopy. The MoS_2_-HS shows the characteristic bands at 377 cm^−1^ and 403 cm^−1^ that are assigned to the in-plane E^1^_2g_ and out of plane A^1^_g_ vibration modes of hexagonal MoS_2_^27^. The presence of the peaks at 448 cm^−1^ and 750 cm^−1^ were assigned to the 2LA(M) and 2E_2g_ vibrational modes of MoS_2_, respectively^36, 37^. The additional peaks at 814 cm^−1^ and 993 cm^−1^ are assigned to the terminal stretching vibration of Mo = O of MoO_3_.^36^ This may be due to the presence of partially oxidised MoS_2_ which was formed after getting exposed to air. In the case of rGO/MoS_2_-S, the characteristic bands for MoS_2_ and rGO were observed and confirmed their presence in the hybrids. Here, the D and G bands of rGO in rGO/MoS_2_-S appears at 1351 cm^−1^ and 1592 cm^−1^, and the I_D_/I_G_ ratio of the composite was calculated to be 1.05. Whereas, the Raman spectrum of pristine rGO exhibits the characteristic D and G bands at 1345 cm^−1^ and 1587 cm^−1^, respectively (Fig. 5, inset) having an I_D_/I_G_ ratio of 1.00^38^.

The XPS analysis was carried out to deduce the exact elemental composition of the rGO/MoS_2_-S (Figure S5). The full scan of the rGO/MoS_2_-S gives the characteristic peaks for Mo3d, S2p, C1s and O1s with their corresponding binding energies. The hi-resolution spectrum of Mo3d has been deconvoluted into four characteristic peaks. The strongest signal at 229.8 and 233 eV represents the characteristic bands of Mo3d_5/2_ and Mo3d_3/2_. Whereas, the signals appeared at 232.8 and 235.6 eV corresponds to the oxidic states of Mo3d_5/2_ and Mo3d_3/2_. That is due to the presence of MoO_3_ or MoO_4_^2−^, which usually resulted in slight oxidation of the MoS_2_ in exposure to atmosphere^39–41^. The analysis of S2p regions shows the characteristic peaks at 163 and 164.9 eV for S2p_3/2_ and S2p_1/2_ of MoS_2_ phase^40^. The deconvoluted peaks of C1s give signals at 284.7 and 286 eV that are assigned to sp^2^ hybridized graphite-like carbon atom and carbon atom bound to one oxygen atom^42^. The weak peaks observed at 287.2 eV is due to the oxygen functional groups of C=O^43^. Similarly the XPS spectra of MoS_2_-HS was carried out to know the presence of elements at their corresponding binding energies (Figure S6). The high-resolution Mo peak of Mo3d gives the characteristic bands of Mo3d_5/2_ and Mo3d_3/2_ at binding energy of 229.8 and 233 eV, respectively. Also, the S2p regions give similar observation like in rGO/MoS_2_-S composite. Further, the specific surface area of as-synthesized materials were estimated by measuring the N_2_ adsorption desorption isotherms at 77 K using the Brunauer-Emmett-Teller method (Figure S7). The rGO/MoS_2_-S hybrid shows higher specific surface area of 104 m^2^/g compared to the pristine MoS_2_-HS (4 m^2^/g) and rGO (19 m^2^/g).

The interesting structure of MoS_2_-HS and its graphene hybrids inspired to study their activity towards energy storage (supercapacitor) and energy conversion (HER) applications. The cyclic voltammetry (CV) and galvanostatic charge-discharge (GCD) techniques were employed in a two compartment three electrodes electrochemical cell to evaluate the electrochemical supercapacitor performance of the as-synthesized materials. Here, the sample modified glassy carbon was taken as the working electrode, Ag/AgCl as the reference and bare platinum wire as the counter electrode in 1 M Na_2_SO_4_ aqueous electrolyte. The overlapped CV and GCD profiles of MoS_2_-HS, rGO and rGO/MoS_2_-S are shown in Figure S8. From CV, the rGO/MoS_2_-S gives humps like a peak at a higher potential and is attributed the pseudocapacitance effect of MoS_2_ to the total capacitance behaviour of the hybrid material. Also, the cycle life of the as-synthesized materials was studied in three electrode system. Interestingly, the rGO/MoS_2_-S shows a retention of 84.4 ± 0.55% of initial capacitance value as compared to the rGO (79.4 ± 0.6%) and MoS_2_-HS (75.8 ± 1%) even after 5000 repeated cycles. That can be ascribed to the synergetic role of both the rGO and MoS_2_ in enhancing the capacitance retention performance of the rGO/MoS_2_-S.

**Figure 6:**
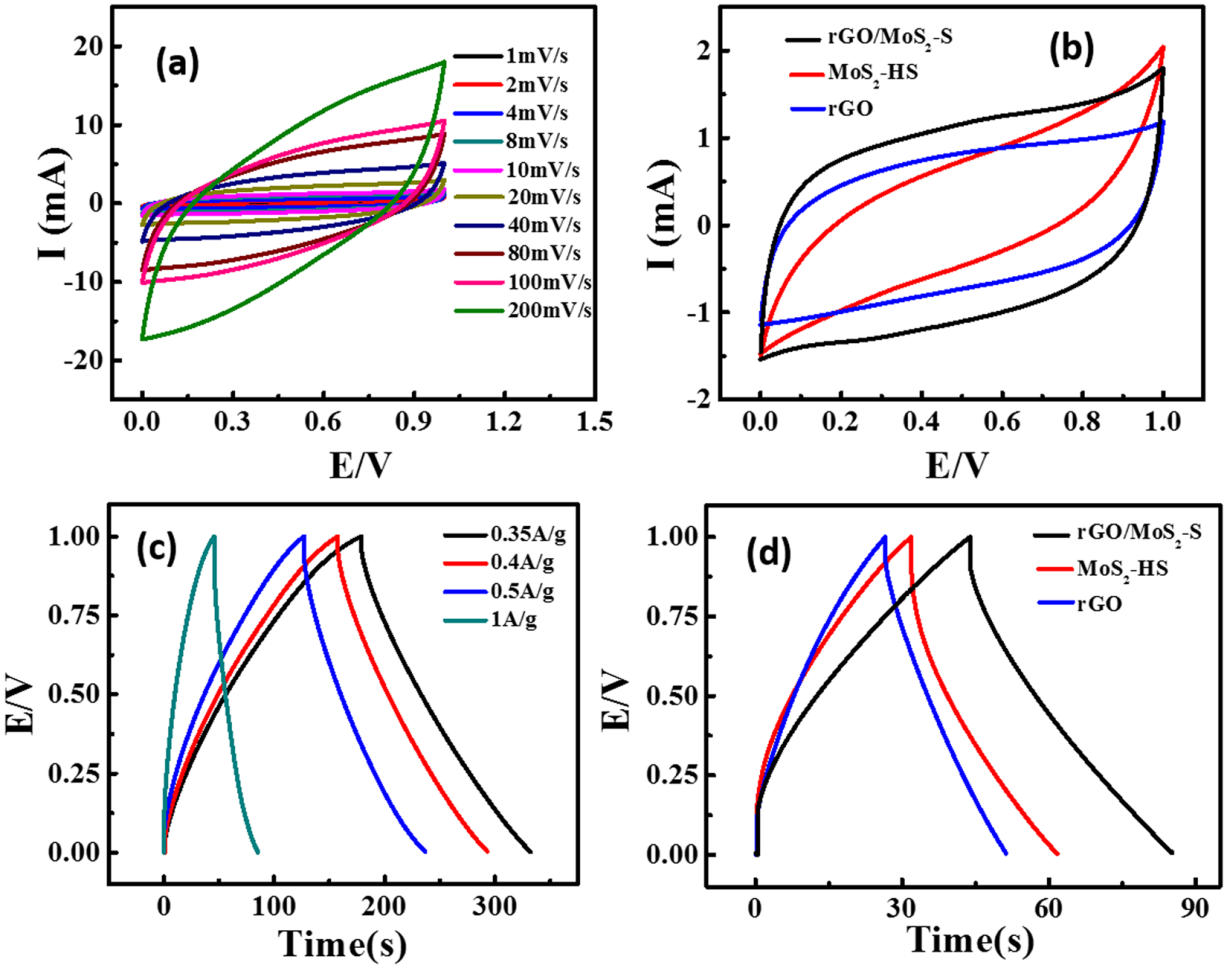
(**a**) CV of rGO/MoS_2_-S hybrid at different scan rate (1 to 200 mV/s), (**b**) CV of MoS_2_-HS, rGO and rGO/MoS_2_-S hybrid at 10 mV/s, (**c**) GCD data of rGO/MoS_2_-S hybrid at different current density and (**d**) GCD of MoS_2_-HS, rGO and rGO/MoS_2_-S hybrid at a current density of 1 A/g.

For the practical application of the as-synthesized material, the electrochemical energy storage performance in a symmetrical two electrode system has been demonstrated. The two electrode design reflects the physical configuration, internal voltages, and capacitance of a packaged supercapacitor^44^. The CV performance of rGO/MoS_2_-S materials at different scan rates are presented in (Fig. 6a). The area under the CV curve shows rectangular and symmetric pattern revealing the better choice for energy storage application. Similarly, the CV performance of MoS_2_-HS and rGO were recorded under the same conditions (Figure S9). The overlapped CV curves of MoS_2_-HS, rGO and rGO/MoS_2_-S are presented in (Fig. 6b). The area under the CV curve of rGO/MoS_2_-S was found to be larger than the rGO and MoS_2_-HS electrode. This observation indicates that the hybrid material shows good capacitive behaviour due to a synergistic contribution of both MoS_2_ and rGO. The specific capacitance of the symmetrical supercapacitor was calculated from CV profile following equation 1.

1 $$Csp=2\times \frac{{\int }_{}^{}I(V)dV}{\nu m{\rm{\bigtriangleup }}V}$$

Where ∫I(V)dV is the area under the CV curve, m is the mass of the electrode materials, ν is the scan rate, and ΔV is the potential window taken. The rGO/MoS_2_-S give highest specific capacitance of 318 ± 14 F g^−1^ compared to rGO (186 ± 12.6 F g^−1^) and MoS_2_-HS (204 ± 17.6 F g^−1^) at a scan rate of 1 mV s^−1^. Here, the hybrids show the capacitance behaviour approximately 1.7 and 1.5 times greater than rGO and MoS_2_-HS electrode material, respectively. Similarly, the galvanostatic charge-discharge (GCD) technique was carried to know the capacitance behaviour of as-synthesized materials at different current densities (Fig. 6c). The GCD curve of rGO/MoS_2_-S shows the symmetric charging and discharging curve. That reveals the good capacitive behaviour of hybrids. The GCD performance of the pristine rGO and MoS_2_ electrodes were carried out for comparison (Fig. 6d). The specific capacitance of the hybrid material was derived from GCD curve following equation 2 ^45^.

2 $${\rm{C}}{\rm{s}}{\rm{p}}=4\frac{I}{{\rm{m}}({\rm{d}}{\rm{V}}/{\rm{d}}{\rm{t}})}$$

Where m is the mass of material taken and dV/dt is the slope of the discharge plot at constant current ‘I’. The specific capacitance of rGO/MoS_2_-S was found to be 190 ± 9.5 F g^−1^ which is higher compared to MoS_2_-HS (97.5 ± 3.5 F g^−1^) and rGO (69.5 ± 2 F g^−1^) at 1 A g^−1^ current density. Here, the unique structure of the rGO/MoS_2_-S material shows better electrochemical behaviour over the rGO and MoS_2_-HS electrode which is validated by both the CV and GCD data. That signifies the synergetic role of rGO and MoS_2_ towards enhancement in capacitor performance of the rGO/MoS_2_-S material. This enhancement could be due to easy intercalation of ions through the Van der Waals gap of MoS_2_ and rGO layers. The presence of rGO in the hybrid material may prevent the agglomeration of the MoS_2_ nanosheets. Therefore, the hybrid possesses more active sites towards the accessibility of electrolyte ion and thereby enhances the capacitance behaviour of the hybrid material. For comparison with available data, the performance of the as-synthesized electrode materials based on MoS_2_ particularly concerning capacitance is summarized in Table S1 ^18, 46–49^. It has been observed that the rGO/MoS_2_-S material has unique characteristics and holds potential for supercapacitor application. The hybrid materials were synthesized at different concentration of GO to explore the effect of rGO content towards the capacitance behaviour. Here, the materials synthesized in the presence of 5 mg, 10 mg and 20 mg of the GO in 30 ml of the reaction solution are referred as rGO/MoS_2_-S(5), rGO/MoS_2_-S and rGO/MoS_2_-S(20), respectively. The CV and GCD measurements of the materials were carried with the similar two electrode system and presented in Figure S10. From CV measurement, It has been observed that the capacitance value increases on increasing the rGO content from rGO/MoS_2_-S(5) to rGO/MoS_2_-S. However, the performance decreased at higher rGO content rGO/MoS_2_-S(20). The GCD measurement reveals the similar performance patterns. So, the rGO content plays the significant role during the growth of rGO/MoS_2_-S and decides the performance towards supercapacitor application.

**Figure 7:**
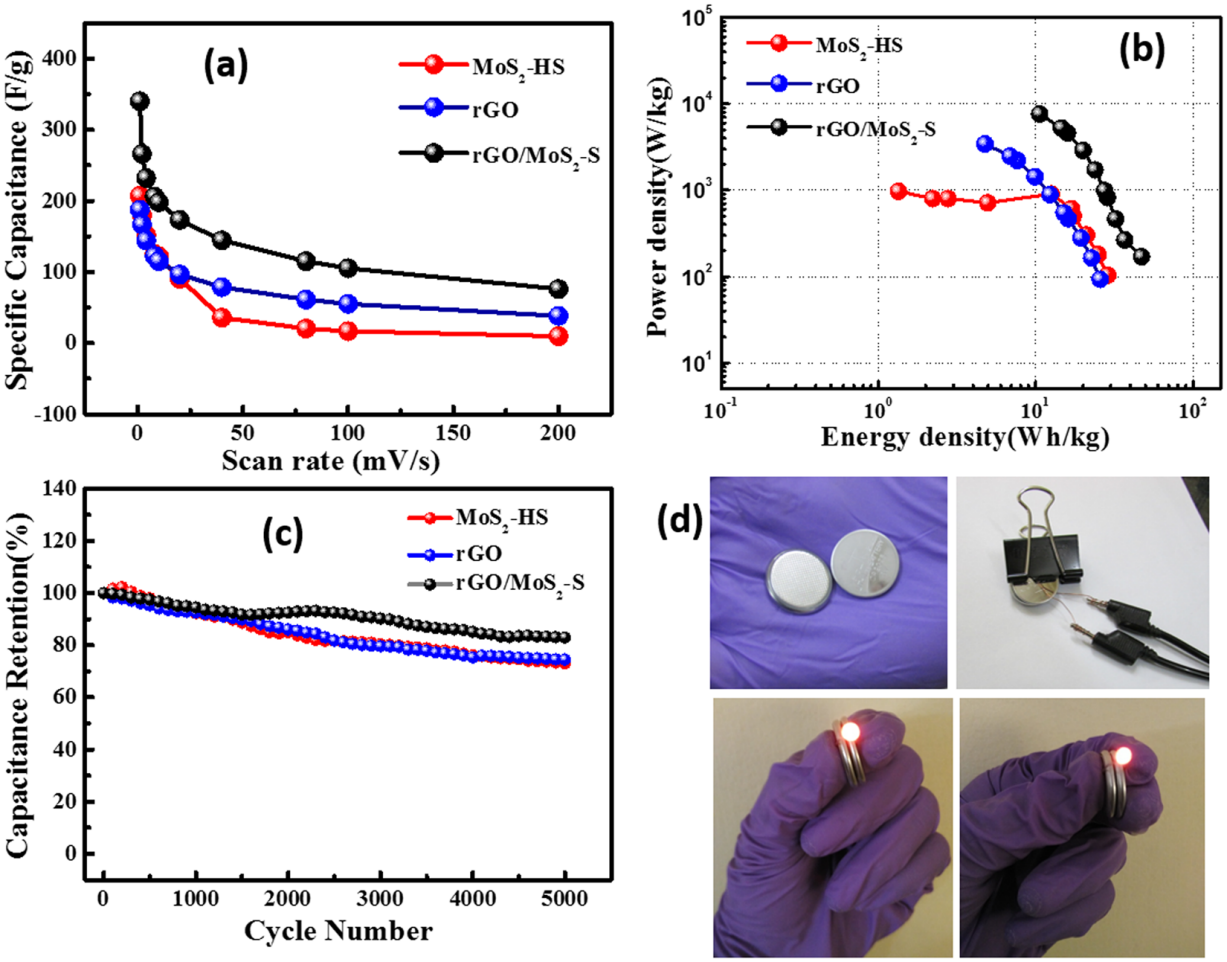
(**a**) Plot of specific capacitance vs. scan rates, (**b**) Ragone plot (energy density vs power density) (**c**) Plot of capacitance retention at different cycles of MoS_2_-HS, rGO and rGO/MoS_2_-S hybrid and (**d**) Photograph demonstrating the powering of a red LED with a stack of two coin cell type supercapacitor device fabricated from rGO/MoS_2_-S hybrid.

The specific capacitance value of the rGO/MoS_2_-S was estimated at different scan rates (Fig. 7a). It has been observed that on increasing the scan rate, the capacitive value decreases. Usually, at higher scan rate, sufficient ion diffusion cannot take place within a constant time, and therefore, reflects a reduction of capacitance values^50, 51^. The energy density (ED) and power density (PD) were calculated from cyclic voltammograms by following the equations 3 and 4 ^52^,

3 $${\rm{E}}.{\rm{D}}.=\frac{1}{2}{\rm{Csp}}{({\rm{\Delta }}{\rm{V}})}^{2}$$

4 $${\rm{P}}.{\rm{D}}.=\frac{1}{2}{\rm{C}}{\rm{s}}{\rm{p}}{({\rm{\Delta }}{\rm{V}})}^{}\nu $$

The Ragone plots (a plot of ED vs. PD) for MoS_2_-HS, rGO and rGO/MoS_2_-S are presented in Fig. 7b. From this plot, the ED and PD values of rGO/MoS_2_-S found to be 44.1 ± 2.1 Wh kg^−1^ and 159.16 ± 7.0 W kg^−1^, respectively. It possesses greater specific energy and power density compared to rGO (25.8 ± 1.7 Wh kg^−1^ and 93.4 ± 6.3 W kg^−1^) and MoS_2_-HS (29.17 ± 1.6 Wh kg^−1^ and 105.33 ± 5.7 W kg^−1^) electrodes, respectively. The long-term cycling stability is one of the most important factors for practical application of a supercapacitor material. Therefore, the cycling stability of the materials has been evaluated using CV technique. Very interestingly, it has been observed that the rGO/MoS_2_-S shows 82 ± 0.95% retention of initial capacitance values compared to MoS_2_-HS (72 ± 1.3%) and rGO (74.1 ± 0.5%) even after 5000 repeated cycles (Fig. 7c). The cycle life of rGO/MoS_2_-S is compared with existing literature and presented in Table S1.

Indeed it is worth to verify the morphology and elemental composition change of the rGO/MoS_2_-S after the capacitance retention test. The post capacitance stability characterisations of rGO/MoS_2_-S was carried by TEM, FESEM, XRD and Raman measurements. The TEM and FESEM images before and after the capacitance retention test are presented in the Figure S11. Interestingly, no such significant change in the morphology of the rGO/MoS_2_-S was observed. That signify the robust nature of the rGO/MoS_2_-S. The post XRD showed the similar diffraction peaks for MoS_2_, but some shift in the 002 plane was observed (Figure S12a). In addition some characteristic peaks appeared for the Na_2_SO_4_ used as the electrolyte. Whereas, the post Raman characterization supports the retention of the characteristic bands for the MoS_2_ and rGO in the hybrids (Figure S12b). But, the peaks at around 814 and 993 cm^−1^ corresponding to the MoO_3_ got decreased. Thus, the characterisations data after capacitance stability test revealed the real phenomena of the as-synthesized materials and guided to improve the performance.

A simple application to power a red light emitting diode (LED) has been demonstrated to validate the potential usefulness of the rGO/MoS_2_-S material based SCs (Fig. 7d). For this purpose, two coin cell supercapacitors (CR2025) were connected in series to extend the potential window for the tandem device. It is clear that the coin cell supercapacitor can power one red LED. The observation and demonstration make a strong case for rGO/MoS_2_-S as a potential supercapacitor material for wider application of interest.

**Figure 8:**
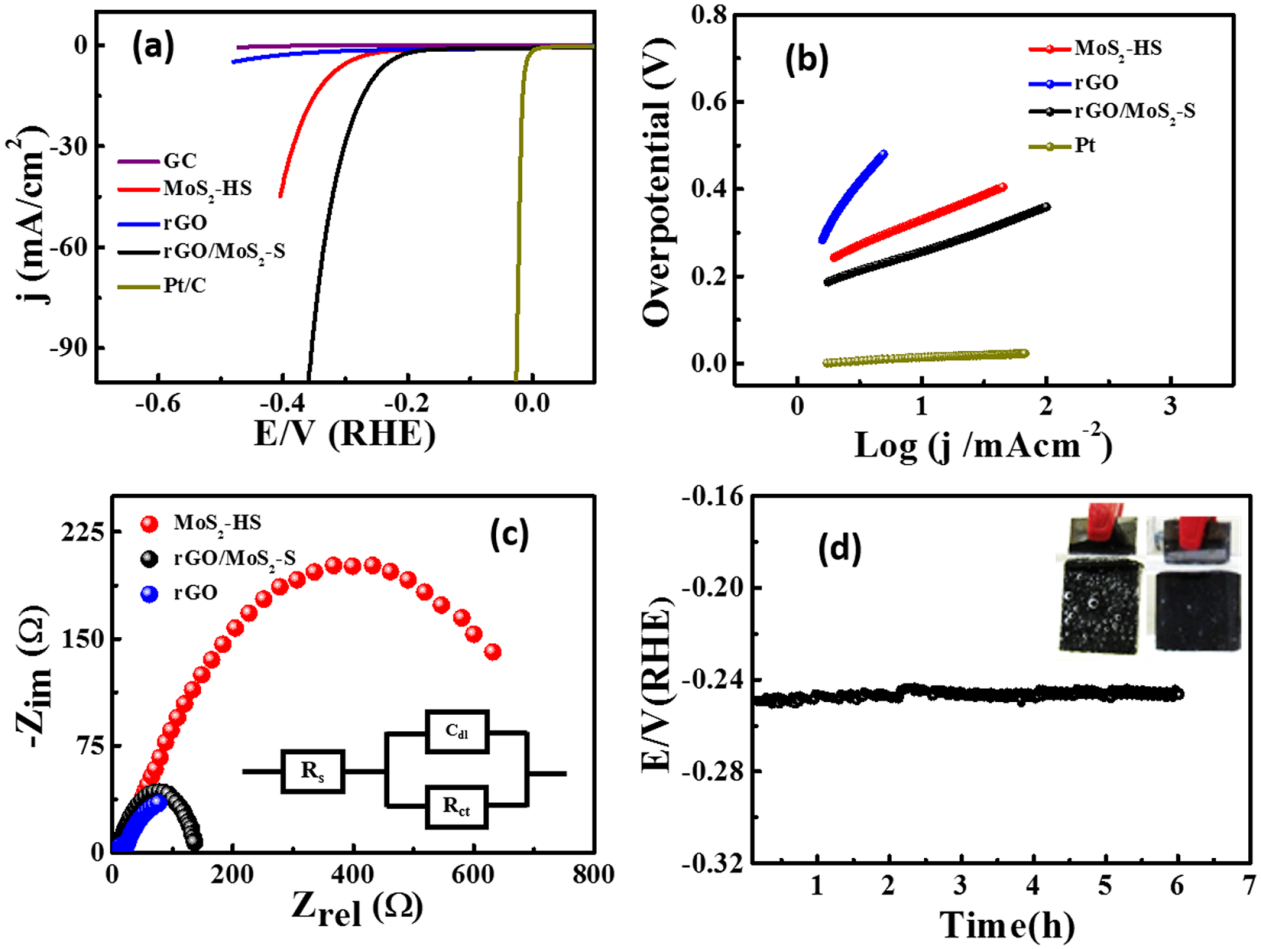
(**a**) Linear sweep voltammograms (LSV) of bare GC, Pt/C, MoS_2_-HS, rGO and rGO/MoS_2_-S hybrid modified electrodes towards HER in 1 M H_2_SO_4_ at a sweep rate of 5 mV/s, (**b**) Corresponding Tafel plots, (**c**) Nyquist plots of MoS_2_-HS, rGO and rGO/MoS_2_-S hybrid modified electrodes.﻿ (**d**) The long-term stability of rGO/MoS_2_-S hybrid at 10 mA/cm^2^ and the inset in (**d**) shows the photograph of the rGO/MoS_2_-S hybrid modified electrode before and during the time of the stability test.

The interesting structure of MoS_2_ and its graphene hybrid material motivated us to investigate the performance for hydrogen evolution. Figure 8a shows the linear sweep voltammetry of the MoS_2_-HS and rGO/MoS_2_-S modified GC electrodes in 0.5 M H_2_SO_4_. The iR compensation has been carried out for all the LSV measurements. The LSV data before and after iR compensation has been presented in Figure S13. The control experiments on bare GC and rGO modified GC were also performed for verification and benchmarked against Pt/C catalyst. It is apparent that the blank GC does not show any HER activity before −0.4 V (vs. RHE) and the rGO too shows no such significant HER activity compared to MoS_2_-HS and rGO/MoS_2_-S. Interestingly, the rapid cathodic current rises beyond −0.145 V in the case of rGO/MoS_2_-S was observed which is indicative of the higher electrocatalytic activity towards the HER. It is surprising that rGO/MoS_2_-S shows higher cathodic current density with a lower onset overpotential of 0.16 ± 0.05 V than the MoS_2_-HS. The rGO/MoS_2_-S yields the state-of-art current density of 10 mA cm^−2^ at an overpotential of 0.25 ± 0.04 V. It shows lower activity compared to the state-of-the-art catalyst, namely Pt/C. However, against the cost and scarcity of Pt, rGO/MoS_2_-S stands out for the lower cost, abundance, and ease of production. The kinetics of the as-synthesized material for HER was investigated by measuring the Tafel slope (Fig. 8b)^53^. The Tafel slope analysis reveals the possible mechanism involved in the HER process. In theory, the mechanism for conversion of H^+^ to H_2_ follows three main paths in acidic medium. In the first step, the electrochemical hydrogen adsorption reaction takes place (H_3_O + e^−^ → H_ads_ + H_2_O) and refers as Volmer reaction. The second step follows the electrochemical desorption path (H_ads_ + H_3_O^+^ e^−^ → H_2_ + H_2_O) and refers as the Heyrovsky reaction. The final step involves the recombination path H_ads_ + H_ads_ → H_2_ and refers as the Tafel reaction. The evolution of hydrogen follows either the Volmer-Heyrovsky or Volmer-Tafel reaction mechanism^54^. Concerning to the previous reports, the Tafel slope value of ≈30 mV dec^−1^, ≈40 mV dec^−1^ and ≈120 mV dec^−1^ represents the Tafel, Heyrovsky and Volmer reactions, respectively as the rate determining step of HER^26^. The Tafel slope of 75 ± 4 mV dec^−1^ for rGO/MoS_2_-S indicate that the reaction proceeds through the Volmer-Heyrovsky mechanism^55^. The rGO/MoS_2_-S exhibits lower Tafel slope compared to the MoS_2_-HS. The enhanced electrocatalytic properties of rGO/MoS_2_-S may be attributed to a strong chemical and electronic interaction between rGO and MoS_2_ which provides the efficient electrical commutation between the catalytic edge sites and the core material. Though the Tafel slope of rGO/MoS_2_-S is higher than that obtained for the benchmarked Pt/C catalyst (30 mV dec^−1^), it showed lower value compared to the previous reports^56^. Further, a comparison was made on the performance of some state-of-the-art catalysts and summarized in Table S2. No doubt rGO/MoS_2_-S finds a place as one of the efficient catalytic materials for HER. Further, the exchange current density (j_0_) of rGO/MoS_2_-S, MoS_2_-HS and Pt/C were calculated from Tafel plot using the extrapolation methods and presented in Figure S14. The rGO/MoS_2_-S exhibits the higher exchange current density (j_0_) of 0.072 ± 0.023 mA cm^−2^ compared to the pristine MoS_2_-HS (0.030 ± 0.025 mA cm^−2^). That signifies the higher activity of the rGO/MoS_2_-S towards HER. It is worth to compare the j_0_ value of the rGO-MoS_2_-S with the MoS_2_ based materials of the previous report. For example, the rGO-MoS_2_-S shows higher j_0_ compared to defect-rich MoS_2_ (8.91 × 10^−3^ mA cm^−2^) ^57^, double-gyroid MoS_2_/FTO (6.9 × 10^−4^ mA cm^−2^)^58^, Core-shell MoO_3_-MoS_2_/FTO (8.2 × 10^−5^ mA cm^−2^)^59^, MoS_3_ particle (6.3 × 10^−7^ mA cm^−2^)^60^, MoS_3_/FTO (1.3 × 10^−7^ mA cm^−2^)^61^, CoS_2_@MoS_2_/RGO (0.0246 mA cm^−2^)^62^, and Cu-MoS_2_/rGO (77.6 × 10^−3^ mA cm^−2^)^63^.

Electrochemical impedance spectroscopy (EIS) measurement was carried out to understand the role of electrode kinetics and interface reaction of MoS_2_ and its graphene hybrid on HER. The Nyquist plots of the samples are presented in Fig. 8c. The observed semi-circle is due to the charge transfer resistance (R_ct_) at the electrode and electrolyte interface. From the impedance fitting, it has been observed that the rGO/MoS_2_-S presents lower charge transfers resistance (R_ct_) of 80 Ω in comparison to MoS_2_-HS (372 Ω). Lower the R_ct_ value of the rGO/MoS_2_-S better is the enhancement in the electron transfer process which goes to enhance HER.

The stability of rGO/MoS_2_-S catalyst for HER was further analysed by Chronopotentiometric electrolysis at a current density of 10 mA cm^−2^ (Fig. 8d). After 6 h of continuous electrolysis, ~6 mV decrease in over potential was observed that indicates a standard level of stability for long term operation. A movie recorded during the hydrogen evolution from the rGO/MoS_2_-S glassy carbon plate electrode is presented in the supporting information (Movie S1). Also, the stability of rGO/MoS_2_-S was carried out with continuous potential cycling the samples over 2000 cycles. The LSV data before and after the potential cycles was recorded (Figure S15a). Interestingly, only a 5 mV difference in the overpotential at the benchmark current density of 10 mA cm^−2^ was observed. That signifies that the rGO/MoS_2_-S is a robust electrocatalyst for HER application to endure from accelerated degradation. Further, the stability of the catalysts before and after the potential cycle was correlated with their before and after cycle EIS measurements (Figure S15b). The R_ct_ values were evaluated from the impedance fitting. The R_ct_ values of rGO/MoS_2_-S before and after stability cycles were estimated to be 80 Ω and 78 Ω, respectively.

Like the capacitance retention test, it is essential to verify the morphology and elemental composition change of the rGO/MoS_2_-S after the HER stability. The post HER characterisations of rGO/MoS_2_-S was carried by TEM, FESEM, XRD, Raman and XPS measurements. The TEM and FESEM images before and after the HER stability test is presented in the Figure S16. Interestingly, no such significant change in the morphology of the rGO/MoS_2_-S was observed. The elemental color mapping spectrum clearly signifies the presence of constituent elements of rGO/MoS_2_-S (Figure S17). That signifies the robust nature of the rGO/MoS_2_-S towards HER. The post-XRD showed the similar diffraction peaks for MoS_2_ but shifting in the (002) plane was observed (Figure S18a). The post-Raman characterization supports the retention of the characteristic bands for the MoS_2_ and rGO in the hybrids (Figure S18b). But, the peaks at around 814 and 993 cm^−1^ corresponding to the MoO_3_ substantially vanished. Further, the XPS data before and after HER stability test is also presented in Figure S19. The full scan of the rGO/MoS_2_-S after HER gives the characteristic peaks for Mo3d, S2p, C1s and O1s with their corresponding binding energies. No such significant change in the peak position was observed. However, an intense peak at 688 eV was appeared and identified as the characteristic peak for F1s. That has been contributed from the Nafion used as a binder for casting the rGO/MoS_2_-S on the electrode surface (experimental section). The deconvolution of high resolution spectra of Mo3d, S2p and O1s was carried out to reveal any changes of the material after HER stability test (Figure S20). No such substantial change in the peak pattern was observed. However, the ratio in the area under the curve for Mo3d and its oxidic peaks was increased. That signifies the reduction of the oxidic states after prolong HER test.

To understand the intrinsic electrocatalytic activity of MoS_2_-HS and rGO/MoS_2_-S, the turnover frequency (TOF) of the catalyst has been evaluated^64^. First, the number of active sites (n) was calculated from the cyclic voltammetry data measured in the potential range of −0.2 to + 0.6V (vs. RHE) in 1 M phosphate buffer (pH = 7) electrolyte at a sweep rate of 50 mVs^−1^ (Figure S21). Here n is directly proportional to the integrated charge (Q_cv_) obtained from the CV measurement and was derived using the equation 5 ^61, 64^.

5 $${\rm{n}}=\frac{{\rm{Qcv}}}{2{\rm{F}}}$$

Where F is the Faraday constant (96485 C mol^−1^). The value of n for MoS_2_-HS and rGO/MoS_2_-S was estimated to be 1.55 × 10^−8^ and 6.8 × 10^−9^, respectively. Thus, the TOF value can be calculated using the equation 6 ^64^.

6 $${\rm{TOF}}=\frac{{\rm{I}}}{2\times {\rm{F}}\times {\rm{n}}}\,$$

Where I, F and n are referred as current (A), Faraday constant (96485 C mol^−1^) and number of active sites, respectively. The TOF was calculated to be 0.623 s^−1^ and 1.47 s^−1^ for MoS_2_-HS and rGO/MoS_2_-S respectively at an overpotential of 250 mV that produces the benchmark current density of 10 mA cm^−2^. At the overpotential of 200 mV s^−1^, the TOF of MoS_2_-HS and rGO/MoS_2_-S was estimated to be 0.107 s^−1^ and 0.340 s^−1^, respectively. It is pertinent to compare the TOF values of rGO/MoS_2_-S with the MoS_x_ based catalyst of previous reports following the above methodology^61, 65, 66^. For example, rGO/MoS_2_-S shows higher TOF compared to amorphous molybdenum plasma pre-treatment (PP) and electrochemical pre-treatment (EP) amorphous molybdenum sulphide on carbon fiber catalysts (0.32, 0.23 and 0.15 s^−1^ for MoSx/PP-CFP, MoSx/EP-CFP and MoSx/N-CFP, respectively at 200 mV)^66^, amorphous molybdenum sulphide film (MoS_3_-CV) catalyst (0.3 s^−1^ at 340 mV). The above observations reveal that the rGO/MoS_2_-S holds certain position concerning higher activity that enhances HER.

In summary, a single-step hydrothermal process has been followed for the synthesis of rGO/MoS_2_-S materials. The electrochemical performance of the hybrid material has been analysed for both the energy conversion and storage applications especially in respect to HER and supercapacitor of interest, respectively. The rGO/MoS_2_-S gives a specific capacitance of 318 ± 14 F g^−1^ with enhanced energy and power density (44.1 ± 2.1 Wh kg^−1^ and 159.16 ± 7.0 W kg^−1^) having capacitance retention of 82 ± 0.95% even after 5000 repeated cycles. Along with the storage efficiency, the rGO/MoS_2_-S shows enhanced performance as a cathode electrocatalyst for HER. It requires only 0.25 ± 0.04 V overpotential to deliver a current density of 10 mA cm^−2^ with a lower Tafel slope of 75 ± 4 mV dec^−1^. That has been supported by a higher TOF value (1.47 ± 0.085 s^−1^) and long term operational stability. The synergistic effect of MoS_2_ and rGO enhances intercalation and transfer process of ions and prevents layered stacking with exposure of additional active edges that are responsible for better energy storage and HER performance. This report provides an interesting method for production of MoS_2_ and its graphene hybrids for potential applications as the electrode material for future energy conversion and storage of general interest.

## Methods

### Materials

Ammonium heptamolybdate, tetrahydrate [(NH_4_)_6_Mo_7_O_24_.4H_2_O, 99.0%], thiourea (NH_2_CSNH_2_, 99%), polyethylene glycol [(PEG)-4000, 99%] were purchased from Himedia, India. The Nafion was procured from Sigma-Aldrich. The deionised (DI) water was obtained by Millipore milli-Q water purification system (18.2 M Ω). All the reagents were directly used without further purification

### Preparation of GO and rGO

The synthesis of graphene oxide was carried out by modified Hummer’s method^67^. Briefly, 1 g of graphite powder was dispersed in 25 ml of concentrated sulphuric acid. Then, 6 g of KMnO_4_ was added slowly to it under ice-cooled condition. Then, it was allowed for continuous stirring up to 2 h at room temperature. Afterward, 50 ml of H_2_O was added under ice-cooled condition followed by slow addition of H_2_O_2_ (30%) till the effervescence of gas seized. The colour of the suspension was changed from brown to yellow. Then, the suspension was filtered and washed with copious amount of 0.1 M HCl and followed by DI H_2_O to remove the SO_4_^2−^ ion. The resulting solid was dried under vacuum and stored for prior use. For the synthesis of reduced graphene oxide (rGO), an aqueous dispersion of GO was made by taking 10 mg of GO in 30 ml of deionised H_2_O followed by 30 minutes of sonication. Then, the aqueous GO dispersion was taken in a 50 ml Teflon-lined stainless steel autoclave. The autoclave was kept at 200 °C for 24 h in an oven for the hydrothermal reduction of GO to rGO. The rGO was collected and dried in a hot air oven and kept it prior to use.

### Synthesis of MoS_2_ hollow spheres (MoS_2_-HS)

MoS_2_-HS was synthesized by a facile one-step hydrothermal process. In this experiment 0.6 g of ammonium heptamolybdate, 0.75 g of thiourea, and 0.7 g of PEG were mixed in 30 ml DI water and sonicated for 45 minutes. Then, the solution was taken in a Teflon-lined 50 ml stainless-steel autoclave and treated at 200 °C for 24 h. The precipitate was collected after cooling down to room temperature and washed with DI water followed by ethanol for several times. The sample was dried in an oven for 12 h at 80 °C and stored in a desiccator for future use.

### Synthesis of rGO and MoS_2_ hybrids (rGO/MoS_2_-S)

The rGO/MoS_2_-S was synthesized in the presence of GO by the same method followed by MoS_2_ hollow sphere synthesis. In brief, 0.01 g of GO dispersion in 30 ml DI H_2_O, 0.6 g of ammonium heptamolybdate, 0.75 g of thiourea and 0.7 g of PEG were mixed with constant stirring for 30 minutes. The solution was transferred to a Teflon-lined 50 ml stainless-steel autoclave and treated at 200 °C for 24 h. After natural cooling to room temperature, the precipitate was collected by centrifugation and washed with DI water followed by ethanol for several times. The sample was dried in an oven for 12 h at 80 °C and stored in a desiccator for future use.

### Electrode preparation

Electrode preparation for supercapacitor testing was carried out with Swagelok type two electrode cell (Figure S22). For this setup, two symmetrical stainless steel electrodes were taken. The homogenous dispersion of well-ground materials sample in ethanol was coated on both the electrodes by drop cast and then dried under vacuum. The cellulose nitrate membrane (of 13 mm diameter) was soaked in a 1 M Na_2_SO_4_ electrolyte solution and used as a separator between the two electrodes. The membrane and materials modified electrode was fitted by Swagelok type setup. The measurements for supercapacitor testing were carried out with Bio-logic Science instrument (VSP-300). The electrochemical performance was also studied by the three-electrode system. For this setup, glassy carbon electrode was used as the working electrode, a platinum wire as the counter, an Ag/AgCl as the reference and 1 M Na_2_SO_4_ used as the electrolyte. In this process for sample loading, 1 mg of as-synthesized samples are separately dispersed in a mixture of 90 µl of ethanol and 10 µl of Nafion (as a binder) sonicated properly to get a homogenous solution. From the above mixture, 10 µl of the solution drop casted on glassy carbon electrode and vacuum dried. Then the electrode was taken for electrochemical study in Bio-logic Science instrument (VSP-300).

The hydrogen evolution reaction was performed with a two-compartment three-electrode cell system containing Platinum wire as the counter electrode, Ag/AgCl as the reference electrode and a glassy carbon (GC, geometrical surface area: 0.07 cm^2^) as the working electrode. In a typical experiment, mixtures of 5% Nafion and as-synthesized samples were prepared and drop casted on GC electrode (of loading 2 mg cm^−2^) followed by vacuum drying. The linear sweep voltammetry was recorded using a computer-controlled Bio-logic science instrument (VSP-300). The electrochemical impedance spectroscopy (EIS) were measured on the same instrument at 1 Hz to 100 kHz, and the data are presented with the Nyquist plot. The potential reported in this report was calculated against the reversible hydrogen electrode (RHE). The potential conversion from Ag/AgCl to RHE was carried out according to equation (7) reported elsewhere^27^.

7 $${E}_{RHE}={E}_{Ag/AgCl}+0.059pH+{E}_{Ag/AgCl}^{0}$$

## Electronic supplementary material

Supporting Information

hydrogen evolution video
