# Supplementary material for: Highly Active 2D Layered MoS2-rGO Hybrids for Energy Conversion and Storage Applications
Source: Sci Rep. 2017 Aug 21;7:8378. doi: 10.1038/s41598-017-08677-5 (PMC5566394; doi:10.1038/s41598-017-08677-5)
Supplement: Supplementary file 1 — Supporting Information [file 41598_2017_8677_MOESM1_ESM.pdf]

Supporting Information

Highly Active 2D Layered MoS_2-rGO Hybrids for Energy Conversion and Storage Applications

Swagatika Kamila,[1,2#] Bishnupad Mohanty,[1,6#] Aneeya K. Samantara,[1,2] Puspendu Guha,[3,4] Arnab Ghosh,[3,5] Bijayalaxmi Jena,[6] Parlapalli V Satyam,[3,4] B. K. Mishra,[1,2] Bikash Kumar Jena[1,2*]

[1] CSIR-Institute of Minerals and Materials Technology, Bhubaneswar-751013, India.

[2] Academy of Scientific & Innovative Research, New Delhi-110001, India.

[3] Institute of Physics, Bhubaneswar-751005, India.

[4] Homi Bhabha National Institute, Training School Complex, Anushakti Nagar, Mumbai 400085, India

[5] Department of Physics, Indian Institute of Technology Kharagpur, Kharagpur 721302, India

[6] Department of Chemistry, Utkal University, Bhubaneswar-751004, Odisha, India

[#]The Authors contributed equally

*Corresponding Author, Email: bikash@immt.res.in**

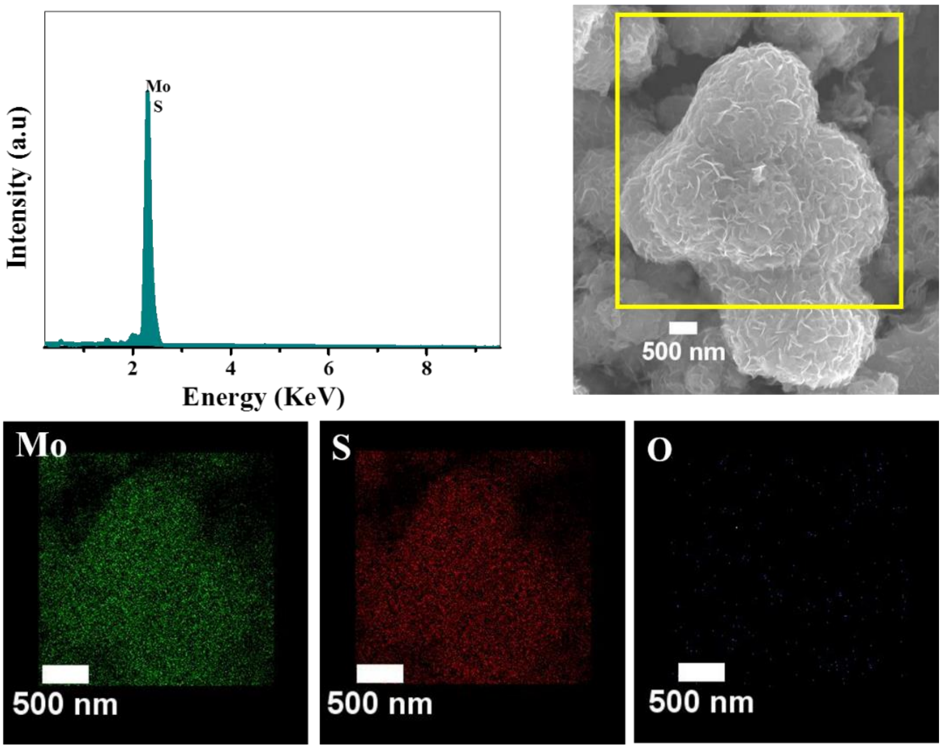

Figure S1: EDS spectrum and color mapping of MoS_2-HS

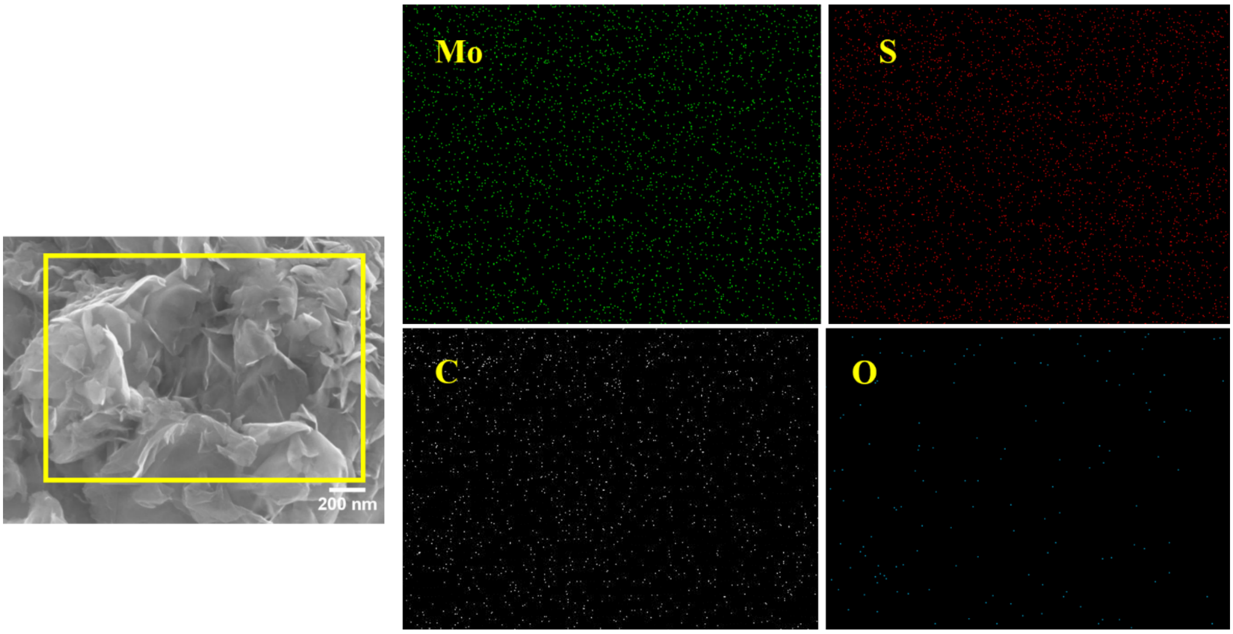

Figure S2: Elemental colour mapping of rGO/MoS_2-S

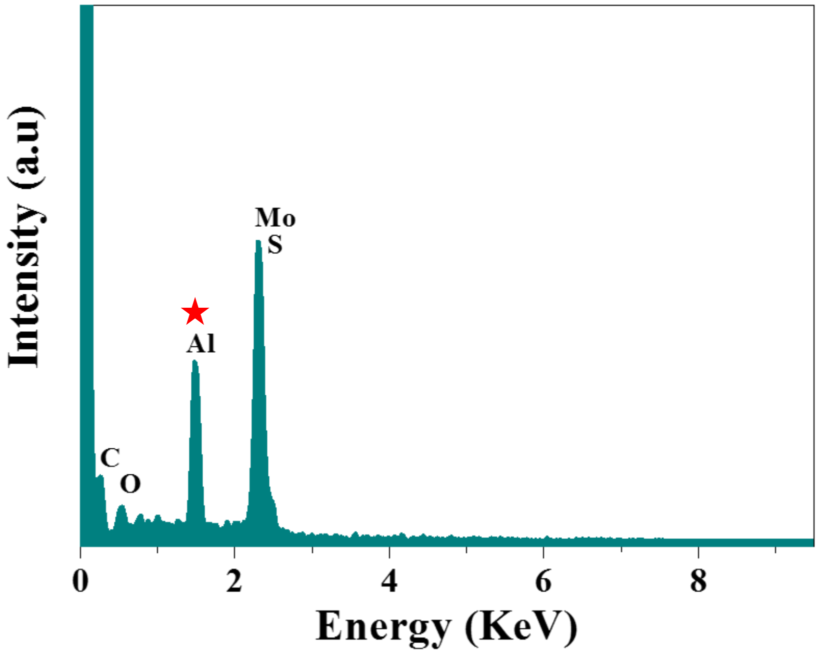

Figure S3: EDS spectrum of rGO/MoS_2-S

★ **The presence of Al is due to the aluminium foil used as substrate for FESEM measurement**

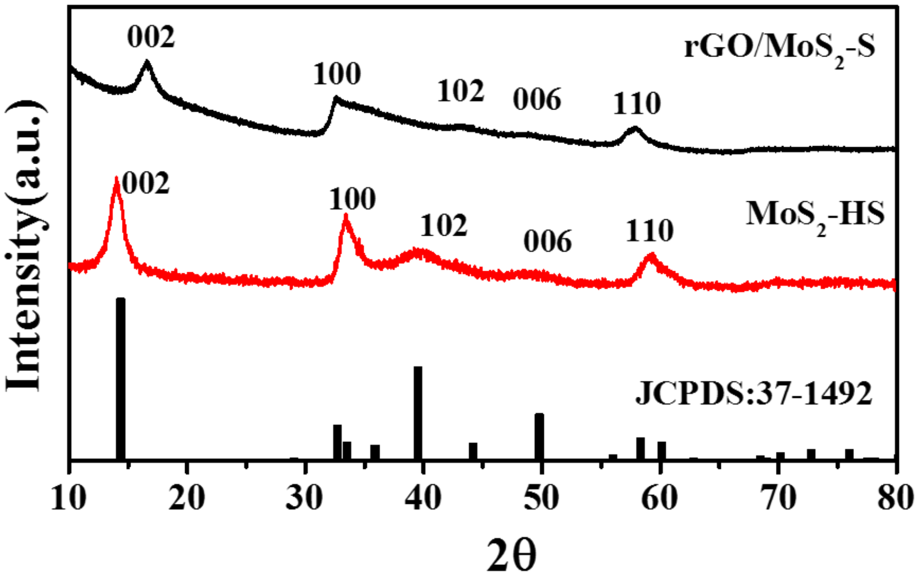

Figure S4: XRD spectrum of MoS_2-HS and rGO/MoS_2-S

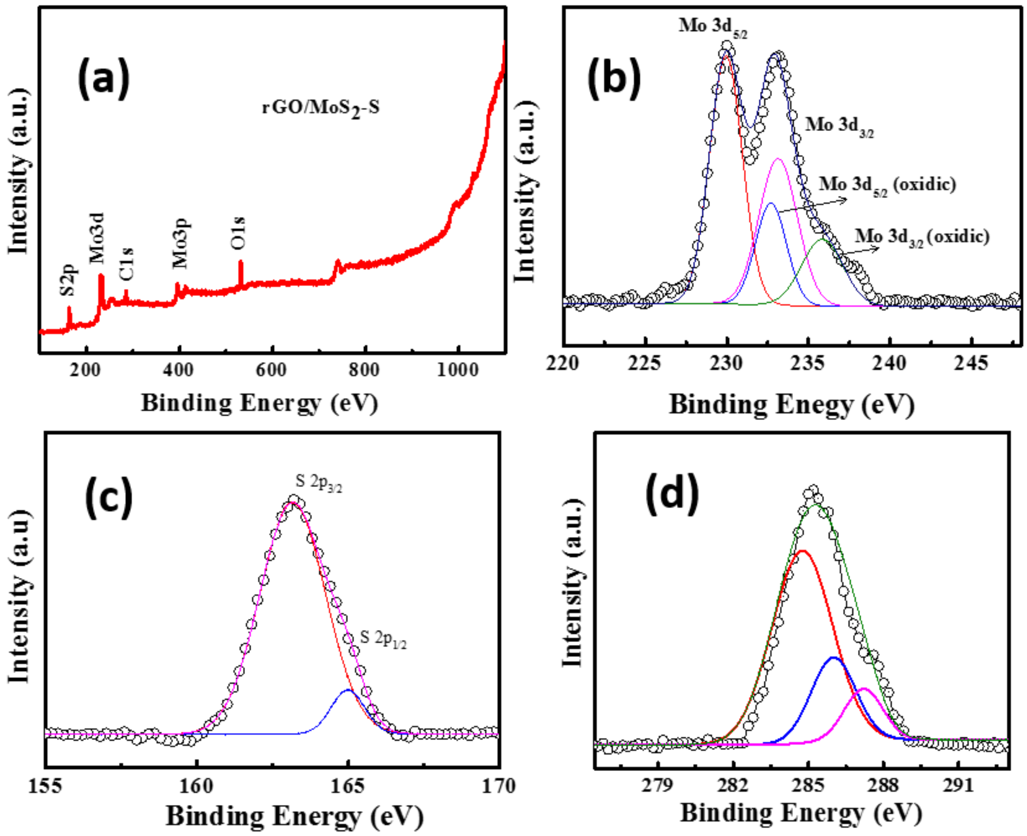

Figure S5: (a) XPS spectrum of rGO/MoS_2-S, high resolution XPS spectra of (b) Mo3d, (c) S2p and (d) C1s

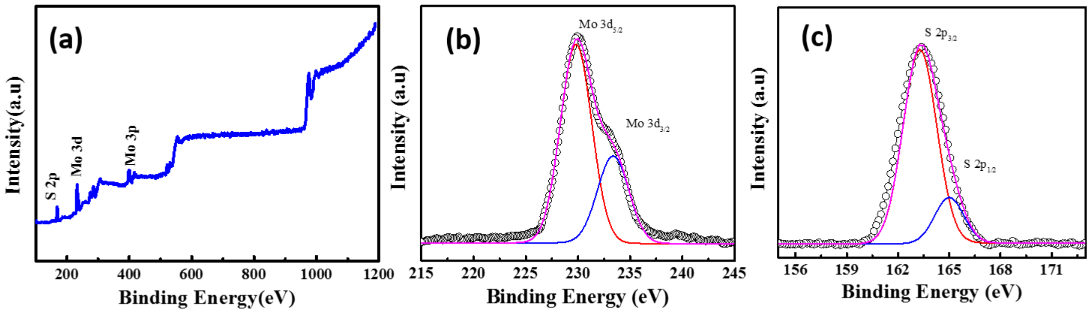

Figure S6: (a) XPS spectrum of MoS_2-HS, high resolution XPS spectra of (b) Mo3d and (c) S2p

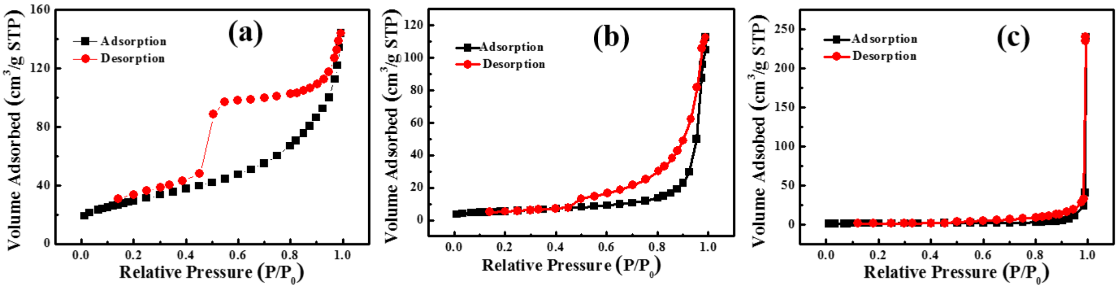

Figure S7: Nitrogen desorption/adsorption isotherm for (a) rGo/MoS_2-S, (b) rGO and (c) MoS_2-HS

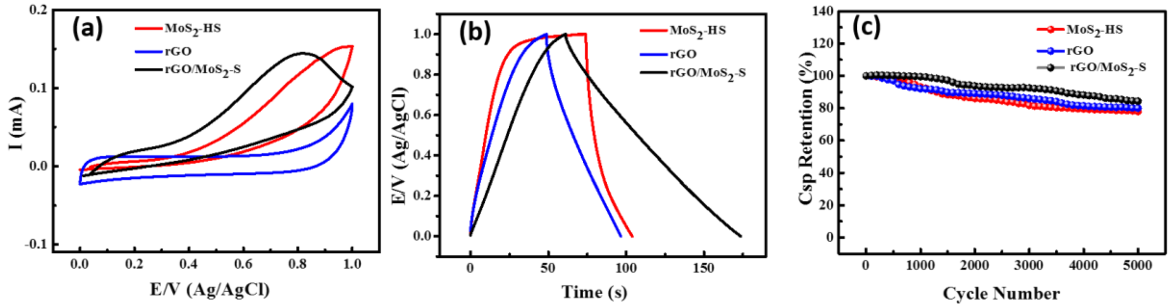

Figure S8: (a) Overlap CV curve at 1mV s^{-1} scan rate, (b) overlap GCD curve at 1A g^{-1} current density and (c) plot of capacitance retention over 5000 cycle of MoS_2-HS, rGO and rGO/MoS_2-S

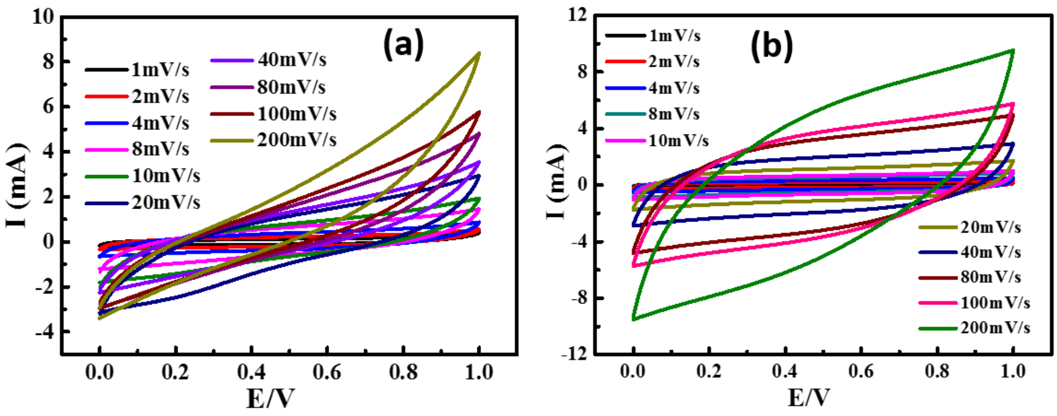

Figure S9: Overlap CV curves of (a) MoS_2 -HS and (b) rGO at different scan rates from 1 mV s^{-1} to 200 mV s^{-1}

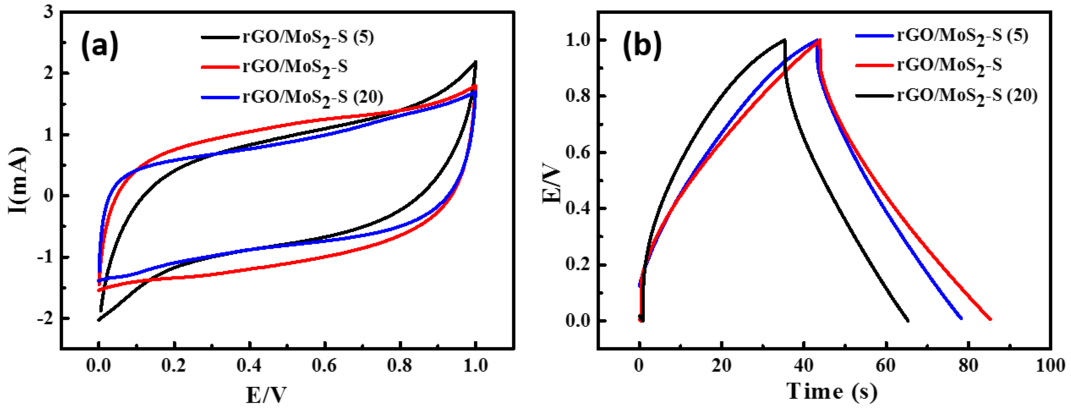

Figure S10: Overlap (a) CV curves at 10 mV s^{-1} scan rate and (b) GCD curves at 1A g^{-1} current density of rGO/MoS_2-S(5), rGO/MoS_2-S and rGO/MoS_2-S(20)

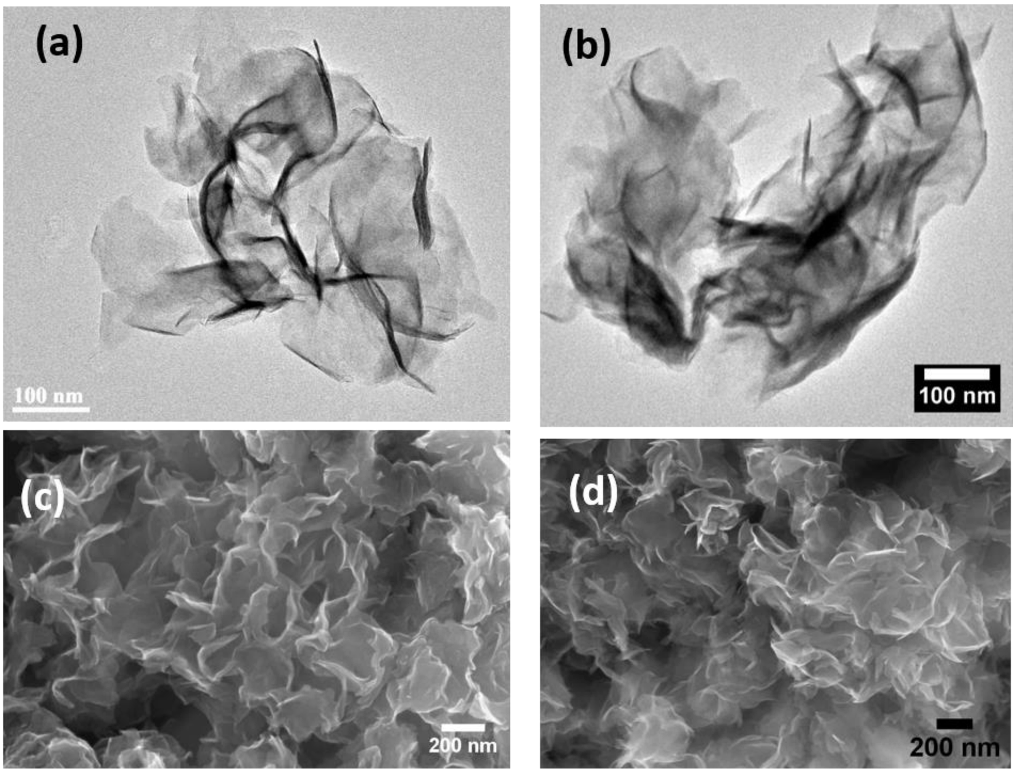

Figure S11: TEM (a, b) and FESEM (c, d) image of rGO/MoS_2-S before (a, c) and after (b, d) capacitance retention test

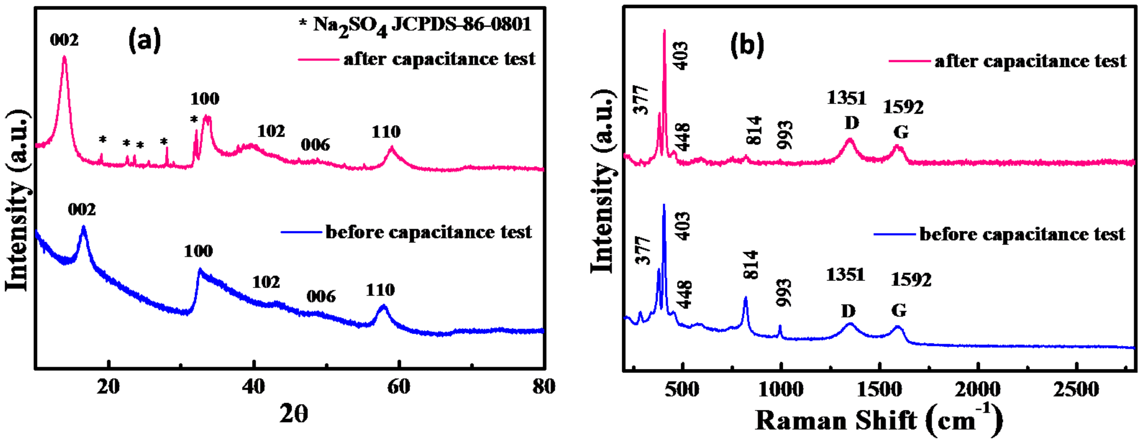

Figure S12: (a) XRD and (b) Raman spectra of rGO/MoS_2-S before and after capacitance retention test

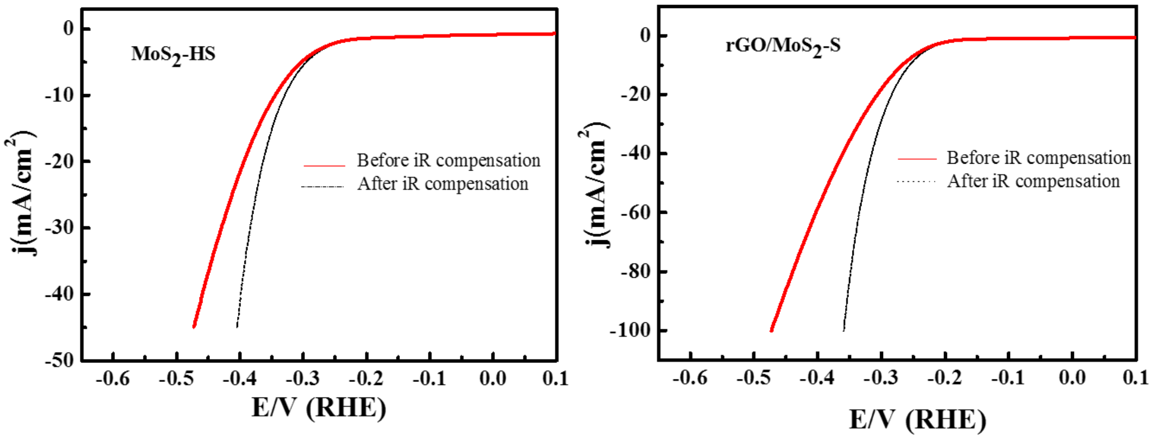

Figure S13: LSV plot before and after iR compensation of MoS_2-HS and rGO/MoS_2-S

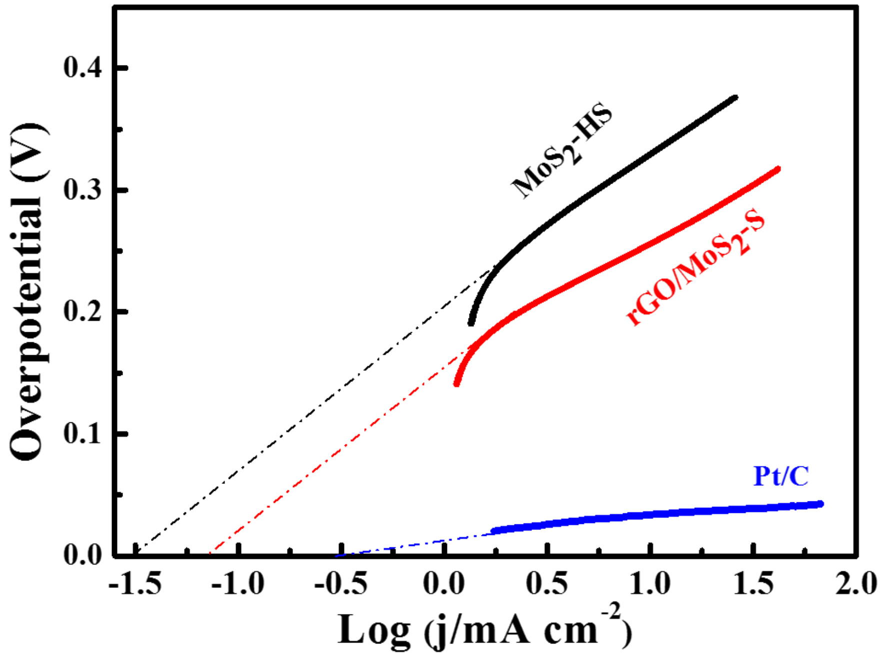

Figure S14: Exchange current density plot derived from Tafel slope of rGO/MoS_2-S, MoS_2-HS and Pt/C

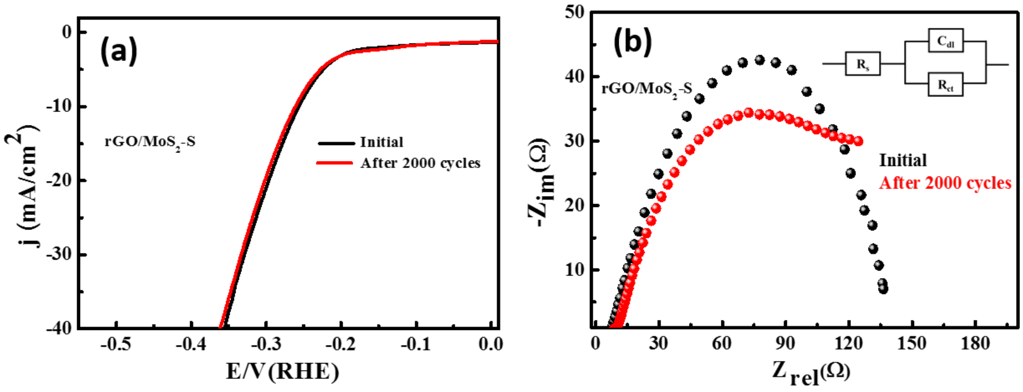

Figure S15: (a) LSV and (b) Impedance spectra of rGO/MoS_2-S before and after 2000 repeated cycles in 0.5M H_2SO_4

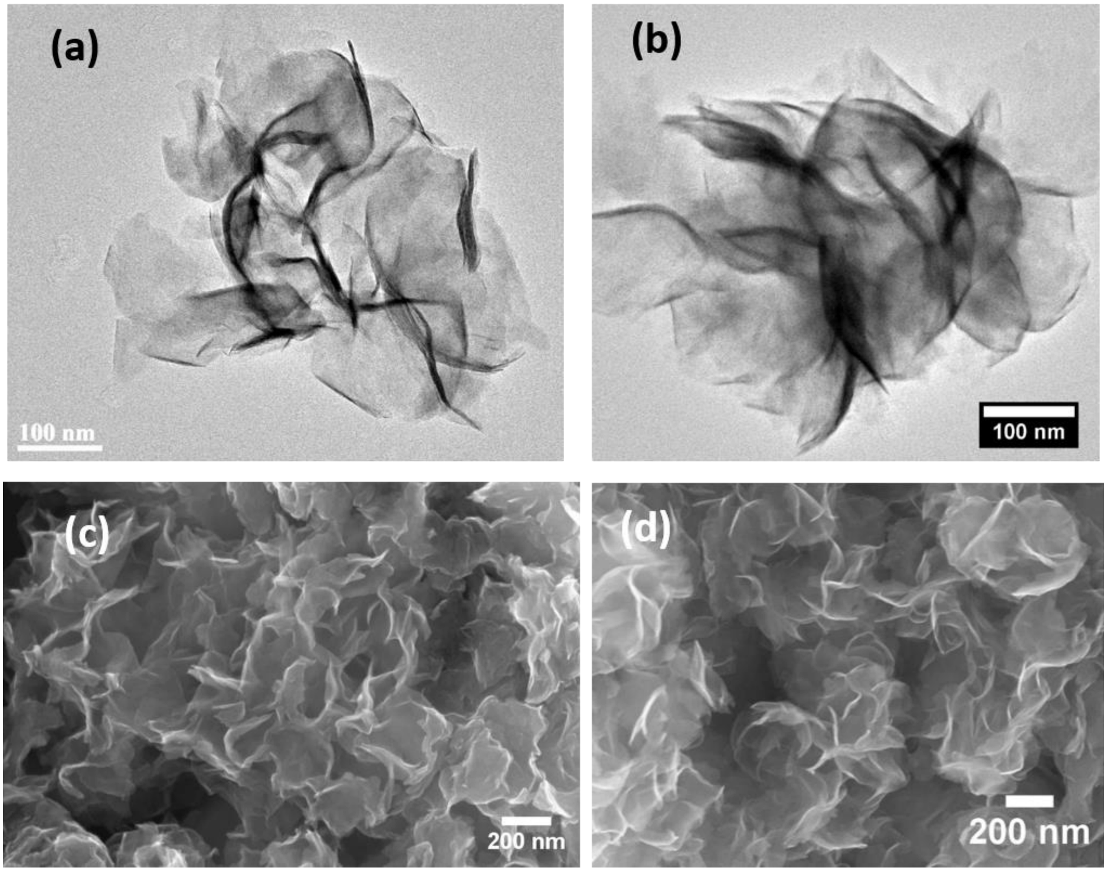

Figure S16: TEM (a, b) and FESEM (c, d) image of rGO/MoS_2-S before (a, c) and after (b, d) HER cycle stability

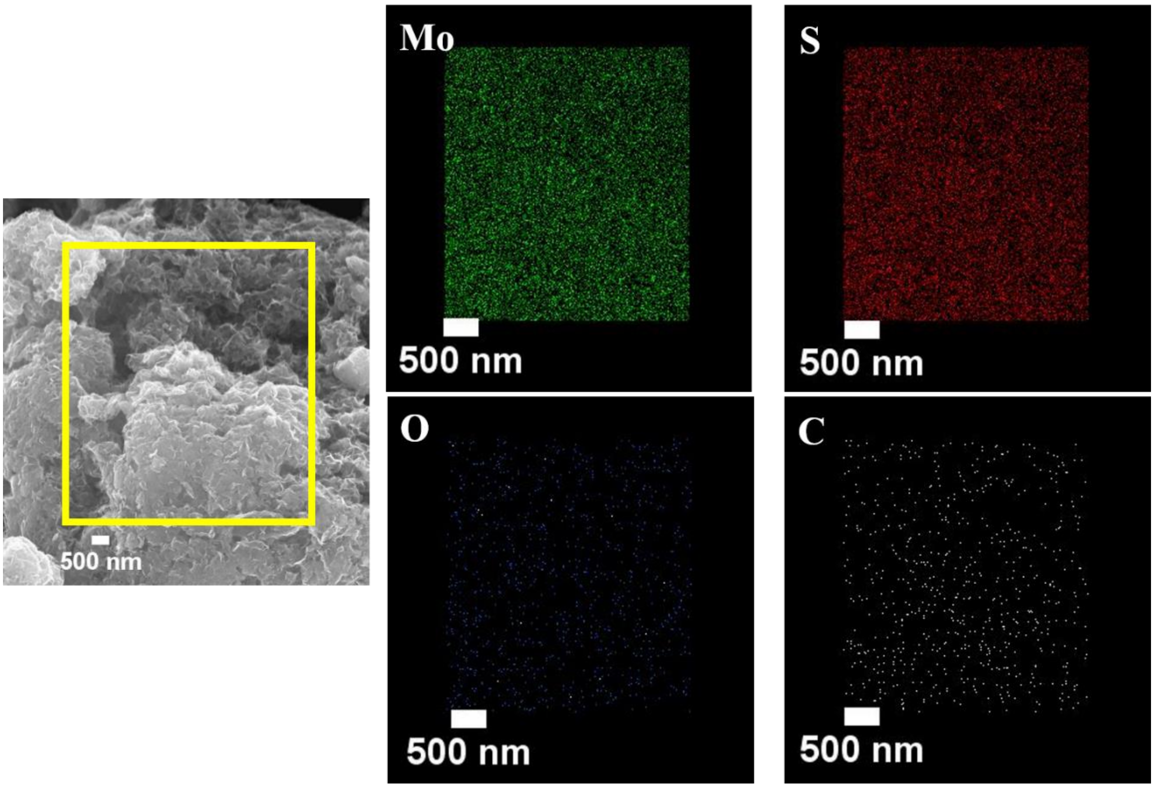

Figure S17: Elemental colour mapping of rGO/MoS_2-S after HER stability in 0.5M H_2SO_4

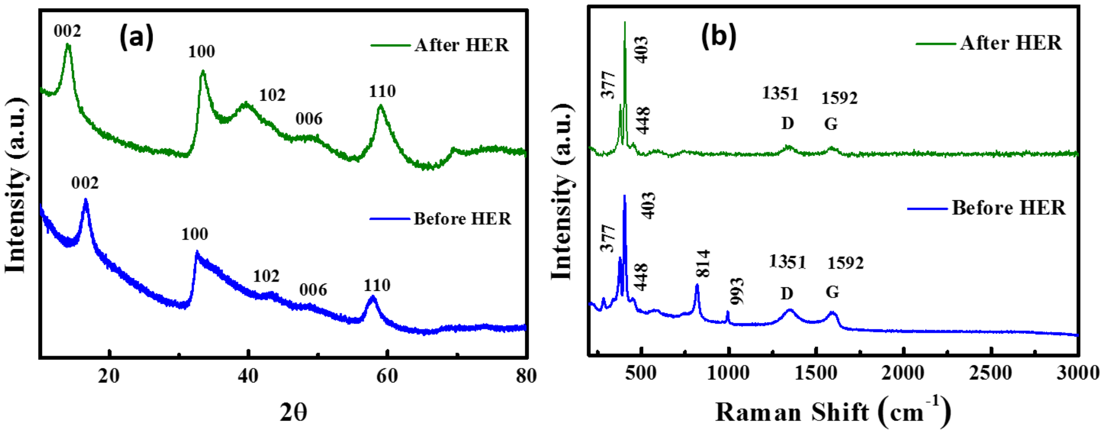

Figure S18: (a) XRD and (b) Raman spectra of rGO/MoS_2-S before and after HER stability

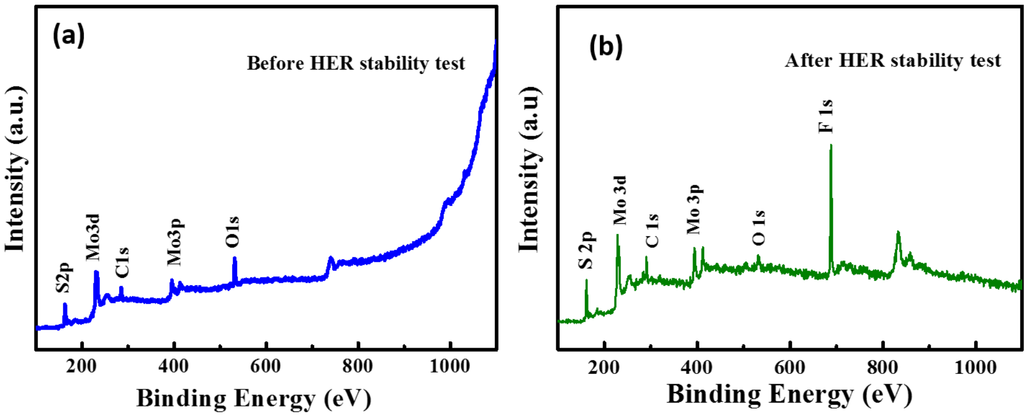

Figure S19: (a) XPS spectrum of rGO/MoS_2-S before and after HER stability

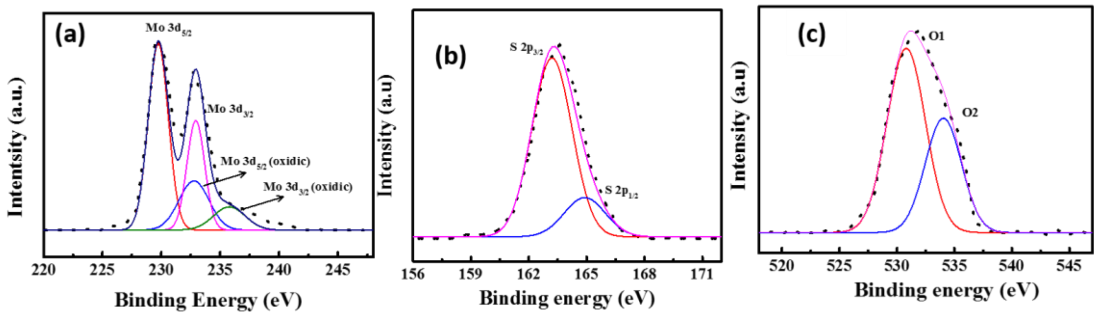

Figure S20: high resolution XPS spectra of (a) Mo3d (b) S2p and (c) O1s of rGO/MoS_2-S after HER stability test

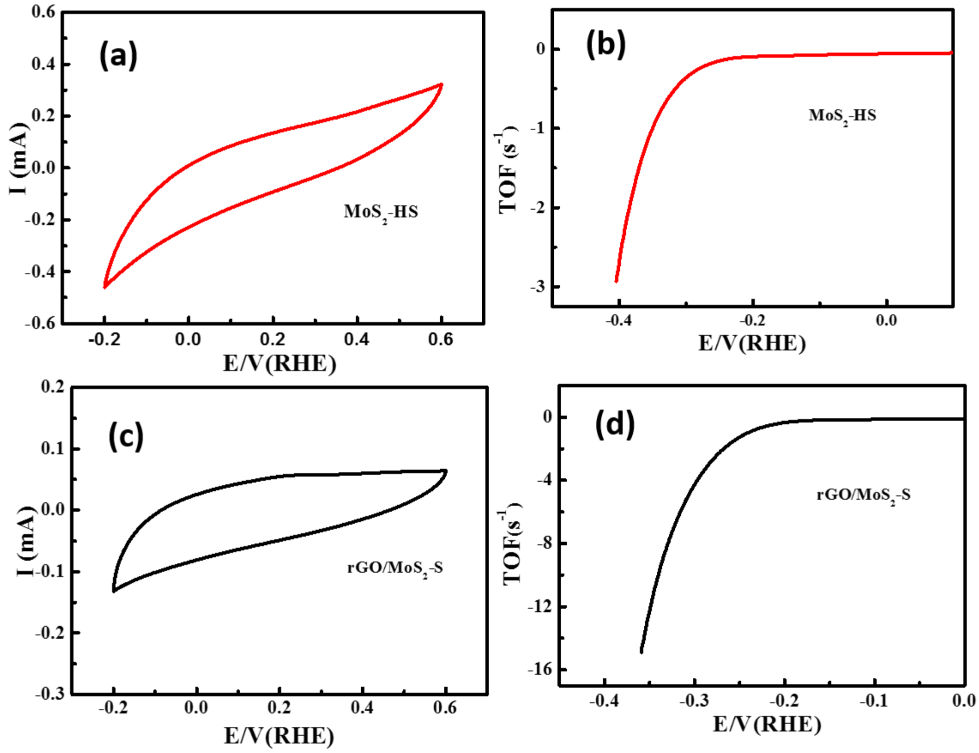

Figure S21: CV of (a) MoS_2-HS, (c) rGO/MoS_2-S in a 1M PBS (pH=7) and corresponding TOF plot for (b) MoS_2-HS and (d) rGO/MoS_2-S

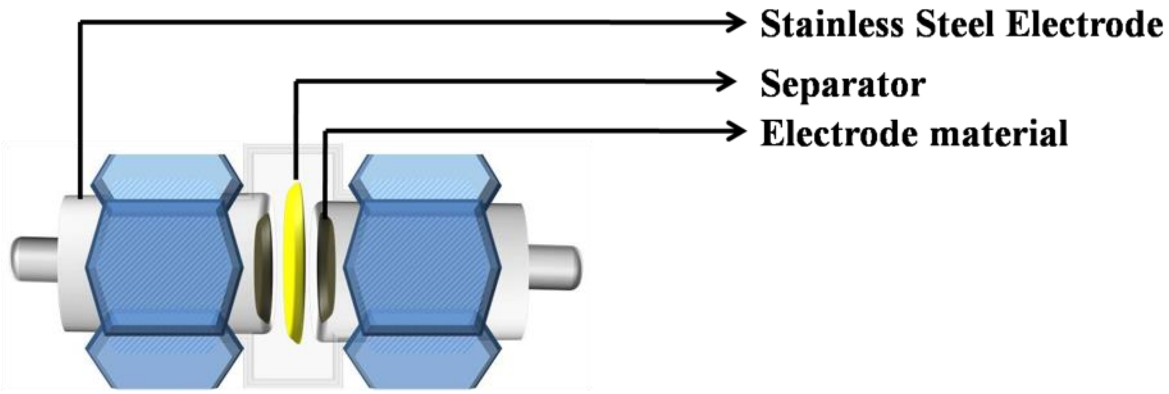

Figure S22: Scheme of symmetrical two electrodes setup

Table S1: Performance comparison of rGO/MoS_2-S with reported materials toward supercapacitor application

**Materials**	**Electrode System**	**electrolyte**	**Specific Capacitance**	**Cycle life**	**Ref**
MoS_2nano sphere	Three	1M Na_2SO_4	106 F/g at 5mV/s	93.8% 1000 cycle	2
MoS_2nanosheet wrapped with Microporous carbon	Three	1M H_2SO_4	189 F/g at 1 A/g	93% 1000 cycle	3
2D MoS_2 on rGO	Three	1M $HClO_4$	265 F/g at 10 mV/s	92% 1000 cycle	4
Layered MoS_2 graphene composite	Three	1M Na_2SO_4	243 F/g at 1 A/g	93.7% 1000 cycle	5
MoS_2-N dop graphene	Three	6M KOH	245 F/g at 0.25 A/g	91.3% 1000 cycle	6
Graphene decorated MoS_2 nanosheet	Three	1M Na_2SO_4	270 F/g at 0.1 A/g	89.6% 1000 cycle	7
MoS_2 graphene aerogel composite	Three	1M Na_2SO_4	268 F/g at 0.5 A/g	93% 1000 cycle	8
N-dop carbon coated MoS_2	Three	1M Na_2SO_4	158 F/g at 0.5 A/g	89% 1000 cycle	9
MoS_2-Co_3O_4 composite	Three	1 M KOH	69 F/g at 0.5 A/g	87% 500 cycle	10
MoS_2@MWCNT	Three	1M Na_2SO_4	452 F/g at 1 A/g	95.8% 1000 cycle	11
MoS_2 carbon composite	Two	1M Na_2SO_4	201 F/g at 0.2 A/g	94.1% 1000 cycle	12
MoS_2 RGO@PANI	Two	1M H_2SO_4	216 F/g at 1 A/g	82% 3000 cycle	13
MoS_2 reduced graphene nano composite	Three	1M KOH	416 F/g at 5mV/s	94.6% 1000 cycle	14
MoS_2/rGO- S	Two	1 M Na_2SO_4	318± 14 F/g at 1mV/s<br>190± 9.5 F/g at 1 A/g	82±0.95% 5000 cycle	Present work

Table S2: Performance comparison of rGO/MoS2-S with reported materials toward HER application

Materials	Onset Potential (mV vs. RHE)	Overpotential (mV) at 10 mA/cm^2	Tafel slope (mV dec^{-1})	Electrolyte	Ref
rGO/MoS_2-S	160	250	72	0.5 M H_2SO_4	Present work
MoS_2 nanoflower/rGO	190	*	95	0.5 M H_2SO_4	15
MoS_2/NGP aerogels	236	261	230	0.5 M H_2SO_4	16
MoS_x/NG	140.6	*	105	0.5 MH_2SO_4	17
MoS_2/rGO	100	156	41	0.5 M H_2SO_4	18
WS_2/rGO	*	265	58	0.5 M H_2SO_4	19
Cu_2MoS_4	*	321	95	0.5M H_2SO_4	20
AS-rich MoS_2 nanosheet	180	220	68	0.5 M H_2 SO_4	21
MoO_3-MoS_2 nanowires	200	254	50~60	0.5 M H_2SO_4	22
MoS_2-nanoflowers	130	275	*	0.50M H_2SO4	23
Ammoniated MoS_2		325	45	0.5M H2SO4	24
Amorphous MoS_x	170-200	160-235	39~63	0.5 M H2SO_4	25
CoMoSx	75	206	78	1M PBS	26
CoWSx	95	271	85	1M PBS	26
NiWSx	165	373	96	1M PBS	26
Double-gyroid MoS_2	150-200	206	50	0.5 MH_2SO_4	27
MoS_2/CNTs	90	184	44.6	0.5 M H2SO_4	28

Sulfur rich MoS_2/SWNT	92	140	41	0.5M H_2So4	29
MoS_2/N-CNT-G	80	*	43.5	0.5M H_2SO_4	30
MoS_2- graphene frame work	121	*	46.3	0.5M H_2SO_4	31
MoS_2/rGO Hydrogel	125	*	41	0.5M H_2SO_4	32
MoS_2/rGO	*	172	43	0.5M H_2SO_4	33
MoS_2/NG	112	*	44	0.5M H_2SO_4	34
MoS_2/rGO Composite	140	*	41	0.5M H_2SO_4	35
MoS_2/G	110	*	41	0.5M H_2SO_4	36
MoS_2/SNG	120	*	45	0.5M H_2SO_4	37
MoS_2 grown on graphene	70	100	41	0.5M H_2SO_4	38

* The data are not available

References:

1. An, L. *et al.* A Self-Standing High-Performance Hydrogen Evolution Electrode with Nanostructured NiCo2O4/CuS Heterostructures. *Adv. Funct. Mater.* **25,** 6814–6822 (2015).
2. Krishnamoorthy, K., Veerasubramani, G. K., Radhakrishnan, S. & Kim, S. J. Supercapacitive properties of hydrothermally synthesized sphere like MoS2 nanostr1. Krishnamoorthy, K., Veerasubramani, G. K., Radhakrishnan, S. & Kim, S. J. Supercapacitive properties of hydrothermally synthesized sphere like MoS2 nanostructures. Mater. . *Mater. Res. Bull.* **50,** 499–502 (2014).
3. Weng, Q. *et al.* Supercapacitive energy storage performance of molybdenum disulfide

nanosheets wrapped with microporous carbons. *J. Mater. Chem. A* **3,** 3097–3102 (2015).

4. da Silveira Firmiano, E. G. *et al.* Supercapacitor Electrodes Obtained by Directly Bonding 2D MoS2 on Reduced Graphene Oxide. *Adv. Energy Mater.* **4,** 1301380 (2014).
5. Huang, K.-J. *et al.* Layered MoS2–graphene composites for supercapacitor applications with enhanced capacitive performance. *Int. J. Hydrogen Energy* **38,** 14027–14034 (2013).
6. Xie, B. *et al.* Hydrothermal synthesis of layered molybdenum sulfide/N-doped graphene hybrid with enhanced supercapacitor performance. *Carbon N. Y.* **99,** 35–42 (2016).
7. Thangappan, R. *et al.* Graphene decorated with MoS2 nanosheets: a synergetic energy storage composite electrode for supercapacitor applications. *Dalt. Trans.* **45,** 2637–2646 (2016).
8. Yang, M., Jeong, J.-M., Huh, Y. S. & Choi, B. G. High-performance supercapacitor based on three-dimensional MoS2/graphene aerogel composites. *Compos. Sci. Technol.***121,** 123–128 (2015).
9. Yang, M., Hwang, S.-K., Jeong, J.-M., Huh, Y. S. & Choi, B. G. Nitrogen-doped carbon-coated molybdenum disulfide nanosheets for high-performance supercapacitor. *Synth. Met.* **209,** 528–533 (2015).
10. Liang, D. *et al.* MoS2 nanosheets decorated with ultrafine Co3O4 nanoparticles for high-performance electrochemical capacitors. *Electrochim. Acta* **182,** 376–382 (2015).
11. Huang, K.-J., Wang, L., Zhang, J.-Z., Wang, L.-L. & Mo, Y.-P. One-step preparation of layered molybdenum disulfide/multi-walled carbon nanotube composites for enhanced performance supercapacitor. *Energy* **67,** 234–240 (2014).

12. Fan, L.-Q., Liu, G.-J., Zhang, C.-Y., Wu, J.-H. & Wei, Y.-L. Facile one-step hydrothermal preparation of molybdenum disulfide/carbon composite for use in supercapacitor. *Int. J. Hydrogen Energy* **40,** 10150–10157 (2015).
13. Li, X. *et al.* Facile Synthesis of MoS2/Reduced Graphene Oxide@Polyaniline for High-Performance Supercapacitors. *ACS Appl. Mater. Interfaces* **8,** 21373–21380 (2016).
14. Gopalakrishnan, K. *et al.* Performance of MoS2-reduced graphene oxide nanocomposites in supercapacitors and in oxygen reduction reaction. *Nanomater. Energy* **4,** 9–17 (2015).
15. Ma, C.-B. *et al.* MoS2 nanoflower-decorated reduced graphene oxide paper for high-performance hydrogen evolution reaction. *Nanoscale* **6,** 5624–5629 (2014).
16. Hou, Y. *et al.* A 3D hybrid of layered MoS2/nitrogen-doped graphene nanosheet aerogels: an effective catalyst for hydrogen evolution in microbial electrolysis cells. *J. Mater. Chem. A* **2,** 13795–13800 (2014).
17. Chen, S., Duan, J., Tang, Y., Jin, B. & Qiao, S. Z. Molybdenum sulfide clusters-nitrogen-doped graphene hybrid hydrogel film as an efficient three-dimensional hydrogen evolution electrocatalyst. *Nano Energy* **11,** 11–18 (2015).
18. Li, Y. *et al.* MoS2 Nanoparticles Grown on Graphene: An Advanced Catalyst for the Hydrogen Evolution Reaction. *J. Am. Chem. Soc.***133,** 7296–7299 (2011).
19. Yang, J. *et al.* Two-Dimensional Hybrid Nanosheets of Tungsten Disulfide and Reduced Graphene Oxide as Catalysts for Enhanced Hydrogen Evolution. *Angew. Chemie Int. Ed.* **52,** 13751–13754 (2013).
20. Tran, P. D. *et al.* Copper molybdenum sulfide: a new efficient electrocatalyst for hydrogen production from water. *Energy Environ. Sci.* **5,** 8912–8916 (2012).
21. Liu, N. *et al.* Microwave-Assisted Reactant-Protecting Strategy toward Efficient MoS2 Electrocatalysts in Hydrogen Evolution Reaction. *ACS Appl. Mater. Interfaces* **7,**

23741–23749 (2015).

22. Chen, Z. *et al.* Core–shell MoO3–MoS2 Nanowires for Hydrogen Evolution: A Functional Design for Electrocatalytic Materials. *Nano Lett.* **11,** 4168–4175 (2011).
23. Wang, D., Pan, Z., Wu, Z., Wang, Z. & Liu, Z. Hydrothermal synthesis of MoS2 nanoflowers as highly efficient hydrogen evolution reaction catalysts. *J. Power Sources* **264,** 229–234 (2014).
24. Wu, Z. *et al.* Enhanced hydrogen evolution catalysis from osmotically swollen ammoniated MoS2. *J. Mater. Chem. A* **3,** 13050–13056 (2015).
25. Merki, D., Fierro, S., Vrubel, H. & Hu, X. Amorphous molybdenum sulfide films as catalysts for electrochemical hydrogen production in water. *Chem. Sci.* **2,** 1262–1267 (2011).
26. Tran, P. D. *et al.* Novel cobalt/nickel-tungsten-sulfide catalysts for electrocatalytic hydrogen generation from water. *Energy Environ. Sci.* **6,** 2452–2459 (2013).
27. Kibsgaard, J., Chen, Z., Reinecke, B. N. & Jaramillo, T. F. Engineering the surface structure of MoS2 to preferentially expose active edge sites for electrocatalysis. *Nat Mater* **11,** 963–969 (2012).
28. Yan, Y. *et al.* Facile synthesis of low crystalline MoS2 nanosheet-coated CNTs for enhanced hydrogen evolution reaction. *Nanoscale* **5,** 7768–7771 (2013).
29. Liu, D. *et al.* Unsaturated-sulfur-rich MoS2 nanosheets decorated on free-standing SWNT film: Synthesis, characterization and electrocatalytic application. *Nano Res.* **9,** 2079–2087 (2016).
30. Li, X. *et al.* Towards free-standing MoS2 nanosheet electrocatalysts supported and enhanced by N-doped CNT-graphene foam for hydrogen evolution reaction. *RSC Adv.* **5,** 55396–55400 (2015).
31. Chang, Y.-H. *et al.* Enhanced Electrocatalytic Activity of MoSx on TCNQ-Treated

Electrode for Hydrogen Evolution Reaction. *ACS Appl. Mater. Interfaces* **6,** 17679–17685 (2014).

32. Zhang, J. *et al.* Three-dimensional MoS2/rGO hydrogel with extremely high double-layer capacitance as active catalyst for hydrogen evolution reaction. *Electrochim. Acta* **182,** 652–658 (2015).
33. Deng, Z. H., Li, L., Ding, W., Xiong, K. & Wei, Z. D. Synthesized ultrathin MoS2 nanosheets perpendicular to graphene for catalysis of hydrogen evolution reaction. *Chem. Commun.* **51,**1893–1896 (2015).
34. Dong, H. *et al.* Three-dimensional Nitrogen-Doped Graphene Supported Molybdenum Disulfide Nanoparticles as an Advanced Catalyst for Hydrogen Evolution Reaction. *Sci. Rep.* **5,** 17542 (2015).
35. Zheng, X. *et al.* Space-Confined Growth of MoS2 Nanosheets within Graphite: The Layered Hybrid of MoS2 and Graphene as an Active Catalyst for Hydrogen Evolution Reaction. *Chem. Mater.* **26,** 2344–2353 (2014).
36. Li, H. *et al.* Charge-Transfer Induced High Efficient Hydrogen Evolution of MoS2/graphene Cocatalyst. *Sci. Rep.* **5,** 18730 (2015).
37. Ren, X. *et al.* MoS2/sulfur and nitrogen co-doped reduced graphene oxide nanocomposite for enhanced electrocatalytic hydrogen evolution. *Int. J. Hydrogen Energy* **41,** 916–923 (2016).
38. Behranginia, A. *et al.* Highly Efficient Hydrogen Evolution Reaction Using Crystalline Layered Three-Dimensional Molybdenum Disulfides Grown on Graphene Film. *Chem. Mater.* **28,** 549–555 (2016).
